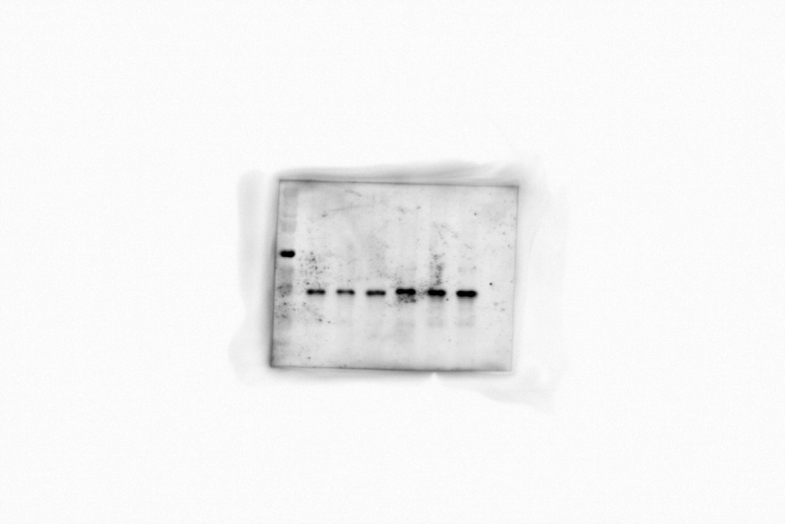# Machine learning deciphers the significance of mitochondrial regulators on the diagnosis and subtype classification in non-alcoholic fatty liver disease

**DOI:** 10.1016/j.heliyon.2024.e29860

**Published:** 2024-04-23

**Authors:** Bingyu Wang, Hongyang Yu, Jiawei Gao, Liuxin Yang, Yali Zhang, Xingxing Yuan, Yang Zhang

**Affiliations:** a Heilongjiang University of Chinese Medicine, Harbin, China; b Department of Gastroenterology, Heilongjiang Academy of Traditional Chinese Medicine, Harbin, China; c Chinese PLA Medical School, Beijing, China; d Zhang Yali Famous Traditional Chinese Medicine Expert Studio, Harbin, China; e Department of Gastroenterology, First Affiliated Hospital of Heilongjiang University of Chinese Medicine, Harbin, China

**Keywords:** Non-alcoholic fatty liver disease, Mitochondrial regulators, Diagnostic biomarker, Machine learning, Subtype classification

## Abstract

**Background:**

Non-alcoholic fatty liver disease (NAFLD) is a highly prevalent liver disease worldwide and lack of research on the diagnostic utility of mitochondrial regulators in NAFLD. Mitochondrial dysfunction plays a pivotal role in the development and progression of NAFLD, especially oxidative stress and acidity β-oxidative overload. Thus, we aimed to identify and validate a panel of mitochondrial gene expression biomarkers for detection of NAFLD.

**Methods:**

We selected the GSE89632 dataset and identified key mitochondrial regulators by intersecting DEGs, WGCNA modules, and MRGs. Classification of NAFLD subtypes based on these key mitochondrial regulatory factors was performed, and the pattern of immune system infiltration in different NAFLD subtypes were also investigated. RF, LASSO, and SVM-RFE were employed to identify possible diagnostic biomarkers from key mitochondrial regulatory factors and the predictive power was demonstrated through ROC curves. Finally, we validated these potential diagnostic biomarkers in human peripheral blood samples and a high-fat diet-induced NAFLD mouse model.

**Results:**

We identified 25 key regulators of mitochondria and two NAFLD subtypes with different immune infiltration patterns. Four potential diagnostic biomarkers (BCL2L11, NAGS, HDHD3, and RMND1) were screened by three machine learning methods thereby establishing the diagnostic model, which showed favorable predictive power and achieved significant clinical benefit at certain threshold probabilities. Then, through internal and external validation, we identified and confirmed that BCL2L11 was significantly downregulated in NAFLD, while the other three were significantly upregulated.

**Conclusion:**

The four MRGs, namely BCL2L11, NAGS, HDHD3, and RMND1, are novel potential biomarkers for diagnosing NAFLD. A diagnostic model constructed using the four MRGs may aid early diagnosis of NAFLD in clinics.

## 1 Introduction

Non-alcoholic fatty liver disease (NAFLD) is a progressive liver disease which characterized by excessive lipid deposition in hepatocytes induced by factors that exclude alcohol and other definite injury [1]. NASH is the progressive period of NAFLD regardless of fibrosis, which is characterized by lobular chronic inflammation and steatosis-induced hepatocellular enlargement and potentially tend to evolve to cirrhosis and hepatocellular carcinoma [2]. Currently, the pathological identification through liver biopsy is the main diagnostic standard for NAFLD. Nevertheless, its high cost and invasiveness make it challenging to apply widely in clinical practice, resulting in the significant under-detection of NAFLD cases [3]. Therefore, exploitation of reliable diagnostic biomarkers for NAFLD attaches importance to the timely diagnosis and intervention whereby improving prognosis.

Mitochondria are essential organelles that maintain the basic cellular functions, including ATP production, fatty acid beta-oxidation, calcium regulation, generation, and elimination of reactive oxygen species (ROS), regulation of apoptosis and activation of the caspase family. The dysfunction of mitochondria is closely related to senescence, carcinogenesis, age-related neurodegenerative diseases, and metabolic syndrome [4,5]. Emerging evidence suggests that mitochondrial dysfunction is a significant contributor to the pathogenesis of NAFLD, although the regulatory mechanisms remain unclear. Currently, the prevailing view is that oxidative stress and fatty acid metabolism disorder are the crucial factors for the development of mitochondrial dysfunction-induced NAFLD [6]. Mitochondrial dysfunction causes fatty acid β-oxidation overload, leading to increased triglyceride synthesis and excessive fat accumulation in hepatocytes, which is one of the typical molecular events of NAFLD. Moreover, mitochondrial dysfunction disrupts the balance between ROS and antioxidants resulting in irreparable cellular damage to hepatocytes, further exacerbating the condition. Hence, mitochondrial dysfunction plays a crucial role in the pathogenesis of NAFLD. Identifying the diagnostic value of mitochondrial regulators could facilitate early detection of NAFLD.

Therefore, we aim to appraise the diagnostic value of mitochondrial associated genes (MRGs) in NAFLD by constructing a diagnostic nomogram in the present study. Potential diagnostic biomarkers for NAFLD identified in four mitochondrial-related hub genes. Furthermore, we successfully validated the expression levels of four mitochondrial related hub genes. This study contributes to a more effective understanding of the heterogeneity and early detection of NAFLD, based on their subtype classification and functional characterization.

## 2 Materials and methods

### 2.1 Data collection

GSE89632 from Gene Expression Omnibus (GEO) database (https://www.ncbi.nlm.nih.gov/geo) was chosen for analysis, which comprised 39 NAFLD liver samples and 24 healthy liver samples. From the MitoCarta3.0 database (http://www.broadinstitute.org/mitocarta), a total of 1136 mitochondrial regulators were obtained.

### 2.2 Recognition of genes expressed differently between NAFLD and healthy

Before data processing, the data were complemented and normalized using impute and limma R packages, respectively. The differentially expressed genes (DEGs) between NAFLD liver samples and healthy liver samples were identified by DESeq2 R package. Healthy liver samples were set as the reference. Genes with |Log_2_FC| > 0.5 and *P* < 0.05 were considered as DEGs. All the DEGs were subsequently submitted for Gene Set Enrichment Analysis (GSEA) functional enrichment analysis using fgsea R package.

### 2.3 WGCNA analysis

With WGCNA R package, we developed a weighted co-expression network based on the transcriptome data in GSE89632. The genes with the highest 25 % absolute deviation from the median were first selected, and then the different gene modules were clustered according to topological overlap. Hierarchical clustering dendrograms were drawn by calculating module feature genes and merging similar modules in the clustering tree. Correlations of different modules with NAFLD and the normal were also analyzed.

### 2.4 Demarcation of key mitochondrial regulators

We intersected DEGs, MRGs, and darkgreen WGCNA gene module thereby obtaining key mitochondrial regulators. Disease Ontology (DO), Gene Ontology (GO), and Kyoto Encyclopedia of Genes and Genomes (KEGG) functional enrichment analyses of these key mitochondrial regulators were conducted by clusterProfiler R package.

### 2.5 Classification of NAFLD subtypes by key mitochondrial regulators

Consensus clustering algorithm is able to determine disease subtypes based on gene expression pattern, which may further help to understand disease heterogeneity. Thus, we employed ConsensusClusterPlus R package to dissect different NAFLD subtypes based on key mitochondrial regulators. CIBERSORT algorithm was used to quantify the infiltration levels of 22 different immune cells of each NAFLD sample. We subsequently compared the immune infiltration pattern between different NAFLD subtypes.

### 2.6 Determination of potential diagnostic biomarkers for NAFLD based on machine learning

In the era of precision medicine, machine learning greatly contributes to the exploitation of novel disease biomarkers [7]. Thus, we applied three machine learning methods to further screen potential diagnostic biomarkers from key mitochondrial regulators. The Random Forest (RF) is a machine learning supervised algorithm based on a decision tree algorithm and used to solve regression and classification problems [8]. The feature importance was determined by the Mean Decrease Gini Index calculated by the RF. The support vector machine recursive feature elimination (SVM-RFE) model was compared with an average misjudgment rate of 10 times cross validation. SVM-RFE is a new machine learning technique that ranks features based on recursion to avoid overfitting [9]. The Least Absolute Shrinkage and Selection Operator (LASSO) regression is a method for reducing dimensionality. It has been shown to outperform regression analysis when evaluating high-dimensional data, and uses regularization to improve prediction accuracy. To perform LASSO analysis, 10-fold cross-validation is recommended by tuning or penalty parameters [10].The randomForest R package, e1071 R package, and glmnet R package were utilized to construct the diagnostic RF model, SVM-RFE model, and diagnostic LASSO model respectively. We then intersected the three models to seek potential diagnostic biomarkers for NAFLD, which turned out to be four MRGs. The differential expression pattern of the four MRGs in NAFLD was investigated. The predictive efficacy of each MRG was demonstrated by diagnostic receiver operating characteristic (ROC) curve. The correlations within the four mitochondrial regulators were further ascertained by Pearson correlation analysis.

### 2.7 Functional enrichment analysis of potential diagnostic biomarkers

Patients with NAFLD were split equally into two groups based on the expression level of each MRGs. GSEA functional enrichment analysis was conducted between low MRG expression group and high MRG expression group, which was considered as the functional characterization of the MRG. We next quantified the levels of diverse functional hallmarkers by single sample Gene Set Enrichment Analysis (ssGSEA) and compared which between healthy liver samples and NAFLD samples. The correlation between the four MRGs and diverse functional hallmarkers were investigated by Pearson correlation analysis.

### 2.8 Construction of the diagnostic nomogram

The four MRGs were subsequently submitted to construct the diagnostic nomogram via rms R package. We carried out a decision curve analysis (DCA) to assess the medical advantage of the diagnostic nomogram.

### 2.9 Clinical samples

The study included 30 NAFLD patients and 30 healthy participants recruited from Heilongjiang Academy of Traditional Chinese Medicine from January 2023 to May 2023. Diagnosis, inclusion, exclusion criteria, and clinical characteristic of the enrolled individuals was shown in Supplementary Fig. 1. We obtained blood samples from the veins and isolated serum by spinning it at 3000 rpm for 20 min. We stored it under refrigerated conditions of 4 °C.

### 2.10 Experimental validation

We decided that 6 mice per group would be sufficient to meet our research objectives, based on a power analysis aiming for 80 % statistical power to detect a significant effect difference with a type I error rate of 5 %, and taking into account ethical and practical considerations. Twelve C57BL/6J mice (18–22 g, male) were obtained from Vital River Laboratory Animal Technology Co., Ltd. (Beijing, China). All mice studied were maintained on a 12 h light/dark cycle at 24 ± 2 °C with free access to food and water. Standard and high-fat diets were bought from the Jiangsu Synergetic Pharmaceutical Bioengineering Co., Ltd. (XTADM001 and XTHF60). The standard diet contains 73.1 % carbohydrates, 4 % fat, and 14.2 % protein, while the high-fat diet contains 20 % carbohydrates, 60 % fats, and 20 % protein. After one week of adaptation, mice were randomized into two groups, the control group (standard diet) and the high-fat diet (HFD) group using a randomized table of numbers according to body mass.

After 16 weeks of feeding, mice were anaesthetised by intraperitoneal injection of 1 % sodium pentobarbital (40 mg/kg) for collection of liver and blood, followed by euthanasia by intraperitoneal injection of 1 % sodium pentobarbital (800 mg/kg). The body weights of the mice were recorded every fortnight during the experimental period.

### 2.11 Histological analysis

For hematoxylin and eosin (Solarbio, G1120) staining, preserve liver tissue in 4 % formaldehyde for 24 h, embedded in paraffin, and then sectioned to 5 μm thick. To stain with oil red O (Solarbio, O8010), frozen liver tissue was sectioned to 15 μm thick and fix at room temperature with 75 % ethanol for 15 min. Subsequently, ORO staining was performed for 1 h, followed by hematoxylin staining. Observe using an optical microscope (CX41, Olympus, Tokyo, Japan). The degree of NAFLD was evaluated by using the NAS score. The NAS scoring system is as follows: Steatosis is scored as 0, 1, 2, and 3 points for less than 5 %, 5 %–33 %, 34 %–66 %, and 67 % or more, respectively; lobular inflammation is scored as 0, 1, and 2 points for less than 2, 2–4, and 4 or more necrotic lesions, respectively; hepatocyte ballooning is scored as 0, 1, and 2 points for no, little, and more ballooning changes, respectively. NAS is scored as the sum of the three scores, ranging from 0 to 8. The NAS score is the sum of the steatosis, lobular inflammation, and hepatocyte ballooning scores and ranges from 0 to 8 [11].

### 2.12 Enzyme-linked immunosorbent assay

Levels of TC (Mlbio, ml037202), TG (Mlbio, ml095894), AST (Mlbio, ml058659), and ALT (Mlbio, ml063179) in the serum were determined using respective kits. Levels of MDA (Jining Shiye, JN24751) and GSH-Px (Jining Shiye, JN20558), and the activity of SOD (Jining Shiye, JN16836) in the liver homogenate were evaluated by using the corresponding kits. We performed the operation according to the manufacturer's instructions.

### 2.13 Western blotting analysis

We obtained protein from liver tissues. Using SDS-PAGE, total protein was resolved in lysis solution and transferred to a PVDF membrane by electroblotting. The membrane was blocked with 5 % skim milk and then incubated with primary antibodies against BCL2L11 (Solarbio, K007923P, 1:1000), NAGS (Proteintech, 21566-1-AP, 1:1000), HDHD3 (Solarbio, K109067P, 1:1000), RMND1 (Abcam, ab223119, 1:1000), and β-actin (Abcam, ab6276, 1:5000) overnight at 4 °C. The membrane was then incubated with a secondary antibody (Abcam, ab6721, 1:5000) diluted to 1:1000 at room temperature for 1 h. Proteins were detected using ECL Plus kits (ThermoFisher, cat.32132) and analyzed using Image-Pro Plus version 6.0 (Vision 6.0, Media Cybernetics, Rockville, MD, USA).

### 2.14 Immunohistochemistry

For semi-quantitative analysis, liver tissues were fixed in 10 % formalin for 24 h at room temperature, embedded in paraffin and cut at 5 μm, followed by incubation with BCL2L11 (Solarbio, K007923P, 1:100), NAGS (Proteintech, 21566-1-AP, 1:100), HDHD3 (Solarbio, K109067P, 1:100), RMND1 (Abcam, ab223119, 1:100) at 4 °C overnight. Sections then were incubated with secondary antibodies (Abcam, ab6721, 1:1000) for 45 min at room temperature. The DAB chromogen was used for incubation, and then hematoxylin was used for counterstaining. Finally, Observation was using an optical microscope (CX41, Olympus, Tokyo, Japan). Using Image-Pro Plus software, protein expression was analyzed as integrated optical density (IOD) in the positive area of the micrograph.

### 2.15 Quantitative real-time PCR analysis

Liver tissue (10 mg) was placed in an EP tube, and 0.2 mL of TRIzol (ThermoFisher, Cat. 15596026) was added. Add 0.2 mL of chloroform, mix well, stand on ice for 3 min and then centrifuge at 12000 rpm for 10 min at 4 °C. The RNA was obtained by adding 400 μL of isopropanol to each tube. The concentration and purity of RNA were determined by a microspectrophotometer (NanoDrop2000, ThermoFisher). The cDNA was obtained by reverse transcription using the Reverse Transcription Kit (ThermoFisher, K1621), and the cDNA was purified by the DNA Purification Kit (Beyotime, D0033). Then, the cDNA was homogenized, and the cDNA concentration of each sample was adjusted to 10 μg/μL. An ABI PRISM 7500 Sequence Detection System (Applied Biosystems, CA, USA) was used to examine the expression levels of the target genes using a standard SYBR Green system. Supplementary Table 2 lists the primer sequences used in this study. The data were analyzed by 2^−ΔΔ^Ct, and GAPDH was used as the internal control.

### 2.16 Statistical analysis

Statistical analysis was conducted using R version 4.0.3 and SPSS version 27.0.1.0. The normality of distribution of variables was assessed by Kolmogorov-Smirnov and Shapiro-Wilk tests. Statistically significance was presented as P value < 0.05. Demographic data analysis was done using either *t*-test or chi-square test. If distribution of data was normal and satisfies homogeneity of variance, student *t*-test was used to compare variables between groups. But if not, Wilcoxon and Mann-Whitney U tests substitute. Correlation was identified by Pearson correlation analysis. |*r*| > 0.1 was considered relevant and P < 0.05 was considered statistically significant. Throughout the study, “*” means P < 0.05, “**” means P < 0.01 and “***” means P < 0.001.

## 3 Results

### 3.1 The DEGs between healthy and NAFLD

We acquired a total of 3064 DEGs, of which 1682 were upregulated in the healthy liver samples and 1382 in the NAFLD samples (Fig. 1A and B). The total DEGs were significantly enriched in peroxisome, base excision repair, steroid hormone biogenesis, DNA replication, ribosome, nucleotide excision repair, bile secretion and nucleotide metabolism, etc. (Fig. 1C). Further functional enrichment analysis revealed that DNA replication, base excision repair, peroxisome, ubiquinone and other terpenoid-quinone biosynthesis, and primary bile acid biosynthesis were significantly strengthened in NAFLD samples, while osteoclast differentiation, IL-17 signaling pathway, rheumatoid arthritis, and TNF signaling pathway were significantly strengthened in healthy liver samples (Fig. 1D and E).

**Fig. 1:**
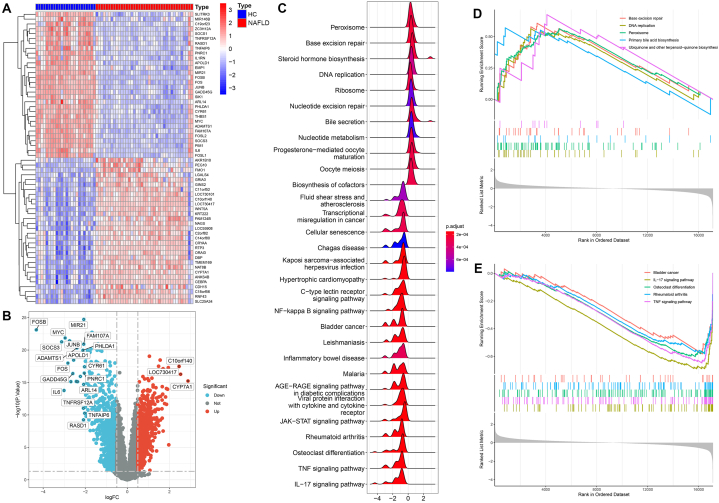
DEGs between healthy liver samples and NAFLD samples. **(A)** Heatmap of the DEGs between healthy liver samples and NAFLD samples. **(B)** Volcano plot of the DEGs between healthy liver samples and NAFLD samples. **(C)** Ridge plot showing the functional characterizations of the entire DEGs. **(D)** Functional strengthens in the NAFLD samples. **(E)** Functional strengthens in the healthy liver samples.

### 3.2 WGCNA gene module associated with NAFLD

A total of 6224 genes were selected for the construction of the gene co-expression network. Twelve gene modules were obtained using a one-step method to construct the co-expression matrix and dynamic hybrid shearing (Fig. 2A). The clustering of the module eigengenes was displayed (Fig. 2B). We also showed the correlation within the twelve gene modules (Fig. 2C). Correlations of the twelve gene modules with NAFLD and the normal were displayed (Fig. 2D). The darkgreen module containing 1197 genes showed the strongest correlation with NAFLD (*r* = −0.86, *P* = 2e-19). We therefore selected the darkgreen module for further analysis.

**Fig. 2:**
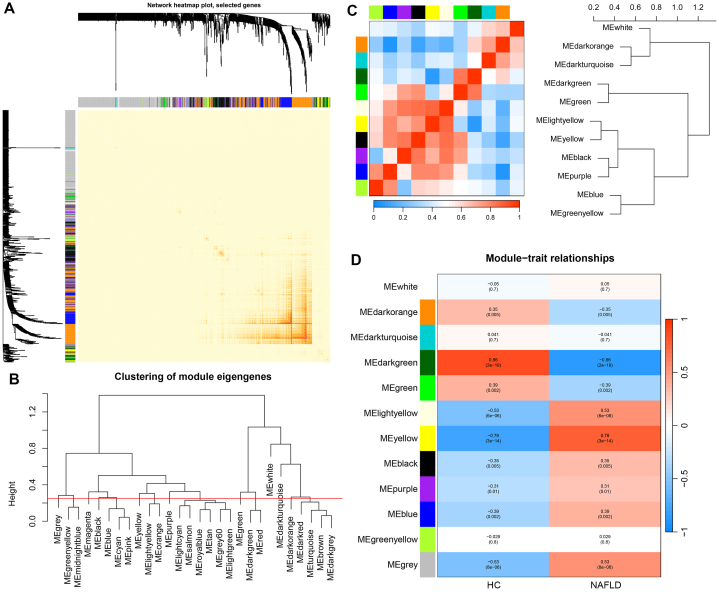
Determination of WGCNA modules. **(A)** Cluster dendrogram of genes with top 25 % median absolute deviation. Each branch indicates a gene and each colour indicates a coexpression module. **(B)** Clustering of module eigengenes. **(C)** Twelve gene module correlations. **(D)** Heatmap of the relationships between modules and characteristics. (For interpretation of the references to colour in this figure legend, the reader is referred to the Web version of this article.)

### 3.3 Identification of key mitochondrial regulators in NAFLD

We intersected the DEGs, MRGs, and darkgreen WGCNA gene module thereby obtaining 25 key mitochondrial regulators (Fig. 3A). GO functional enrichment analysis suggested that these key mitochondrial regulators were significantly enriched in regulation of oxidoreductase activity, mitochondrial transport, regulation of mitochondrion organization, mitochondrial inner membrane, mitochondrial matrix, mitochondrial outer membrane, adenine nucleotide transmembrane transporter activity, and ATP transmembrane transporter activity, etc. (Fig. 3B). DO functional enrichment analysis showed that these key mitochondrial regulators mainly participated in renal carcinogenesis and thyroid disease (Fig. 3C). KEGG functional enrichment analysis suggested that these key mitochondrial regulators were significantly enriched in peroxisome, FOXO signaling pathway, biosynthesis of cofactors, and tuberculosis, etc. (Fig. 3D).

**Fig. 3:**
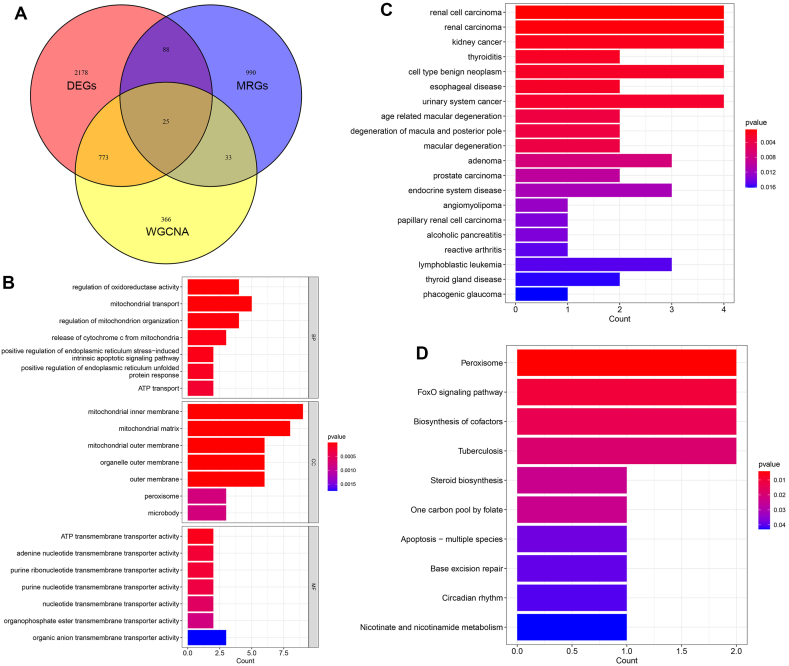
Identification of key mitochondrial regulators. **(A)** Intersection of DEGs, MRGs, and darkgreen WGCNA gene module. **(B)** Functional enrichment analysis of the key mitochondrial regulators using GO was conducted. **(C)** Functional enrichment analysis of the key mitochondrial regulators using DO was conducted. **(D)** Functional enrichment analysis of the key mitochondrial regulators using KEGG was conducted.

### 3.4 Classification of NAFLD subtypes

We determined two distinct NAFLD subtypes by consensus clustering, which were named cluster 1 (C1) and cluster 2 (C2) (Fig. 4A-C). Next, the proportion of 22 different immune infiltrating cells of each NAFLD sample was quantified (Fig. 4D). It turned out to be that the infiltration level of γδ T cells was higher in C2, whereas the infiltration level of mast cells activated was more abundant in C1 (Fig. 4E).

**Fig. 4:**
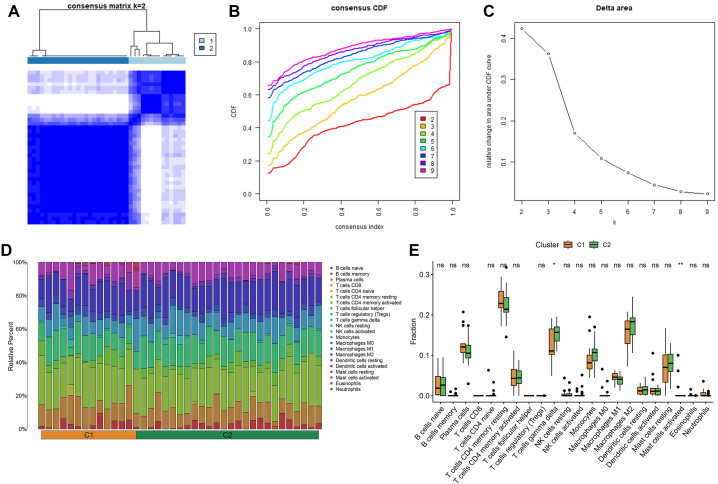
Key mitochondrial regulators are used to classify NAFLD subtypes. **(A**–**C)** Consensus clustering. **(D)** Quantifying the levels of infiltration of 22 immune cells in each sample of the NAFLD. **(E)** Comparison of the immune infiltration pattern between different NAFLD subtypes.

### 3.5 Potential diagnostic biomarkers for NAFLD

The SVM-RFE, LASSO, and RF machine learning models based on key mitochondrial regulators were constructed respectively (Fig. 5A-C). We further found that four MRGs (BCL2L11, NAGS, RMND1, and HDHD3) were overlapped among the three models, which were finally considered as the potential diagnostic biomarkers for NAFLD (Fig. 5D). Correlation analysis showed that BCL2L11 significantly negatively correlated with the other three MRGs, while NAGS, RMND1, and HDHD3 significantly positively correlated with each other (Fig. 5E).

**Fig. 5:**
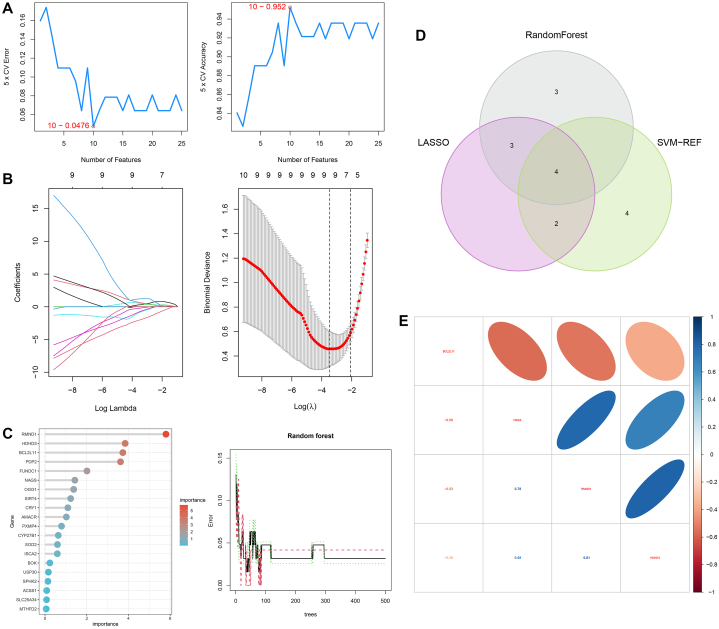
Determination of potential diagnostic biomarkers for NAFLD. **(A)** Construction of the SVM-RFE model. **(B)** Construction of the diagnostic LASSO model. **(C)** Construction of the diagnostic RF model. **(D)** Determination of potential diagnostic biomarkers for NAFLD. **(E)** Correlations within the four diagnostic biomarkers.

**Fig. 6:**
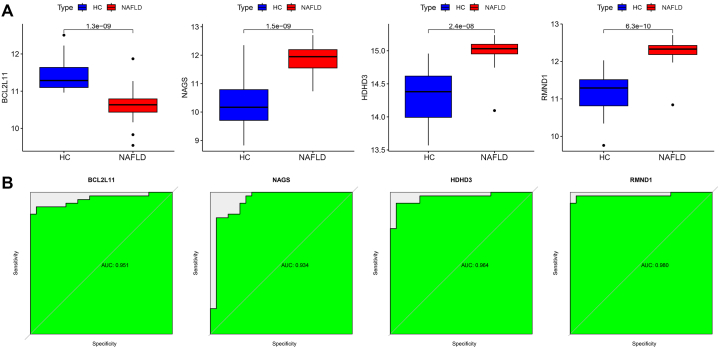
The differential expression and predictive efficacy of the four potential diagnostic biomarkers. **(A)** The differential expression of the four potential diagnostic biomarkers between NAFLD samples and healthy liver samples. **(B)** ROC curves showing the predictive efficacy of the four potential diagnostic biomarkers.

We subsequently verified that the differential expression of the four potential diagnostic biomarkers between NAFLD samples and healthy liver samples (Fig. 6A). BCL2L11 was significantly downregulated in NAFLD, whereas NAGS, RMND1, and HDHD3 were significantly upregulated in NAFLD. We assessed the predictive efficacy of the four potential diagnostic biomarkers by ROC curves. The AUCs of BCL2L11, NAGS, RMND1, and HDHD3 were 0.951, 0.934, 0.980, and 0.964 respectively (Fig. 6B).

### 3.6 Functional characterization of key mitochondrial regulators

**Fig. 7:**
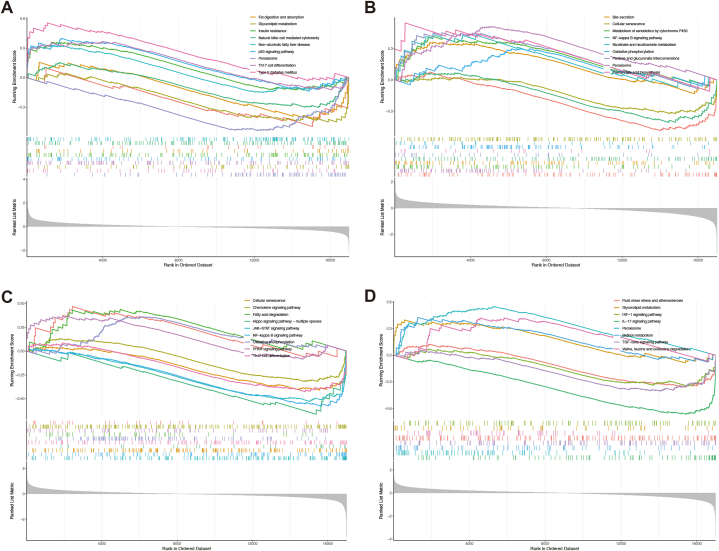
Functional characterization of the four potential diagnostic biomarkers. **(A)** Functional characterization of BCL2L11. **(B)** Functional characterization of NAGS. **(C)** Functional characterization of HDHD3. **(D)** Functional characterization of RMND1.

GSEA functional enrichment analysis suggested that BCL2L11 may associate with fat digestion and absorption, glycerolipid metabolism, insulin resistance, peroxisome, and th17 cell differentiation (Fig. 7A). NAGS may associate with bile secretion, cellular senescence, metabolism of xenobiotics by cytochrome P450, oxidative phosphorylation, and pentose and glucuronate interconversions (Fig. 7B). HDHD3 may associate with fatty acid degradation, Hippo signaling pathway-multiple species, JAK-STAT signaling pathway, oxidative phosphorylation, and PPAR signaling pathway (Fig. 7C). RMND1 may associate with glycerolipid metabolism, HlF-1 signaling pathway, IL-17 signaling pathway, retinol metabolism, and valine, leucine and isoleucine degradation (Fig. 7D).

**Fig. 8:**
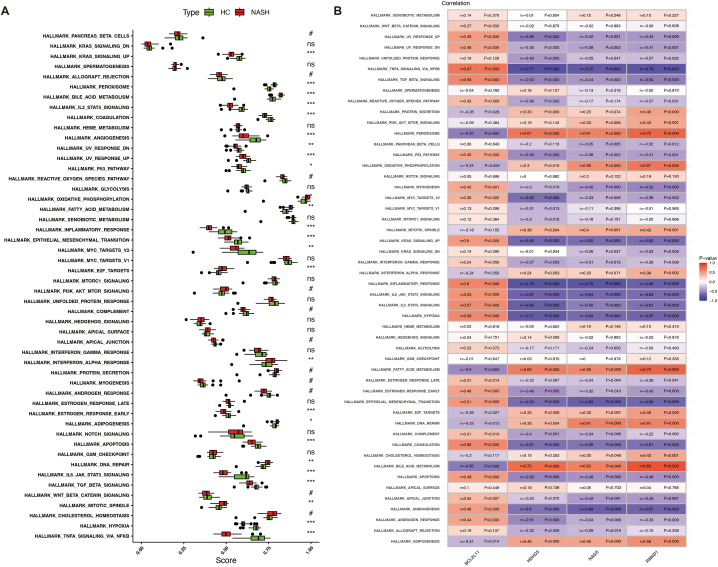
Correlations between the four potential diagnostic biomarkers and diverse functional hallmarkers. **(A)** The difference of diverse functional hallmarkers between NAFLD samples and healthy liver samples. **(B)** Correlations between the four potential diagnostic biomarkers and diverse functional hallmarkers.

Diverse functional hallmarkers were found to be aberrantly regulated in NAFLD. DNA repair, IFN-α response, fatty acid metabolism, peroxisome, and bile acid metabolism, etc. were significantly strengthened in NAFLD, while KRAS signaling upregulation, IL2-STAT5 signaling, angiogenesis, P53 pathway, and inflammatory response, etc. were significantly weakened in NAFLD (Fig. 8A). Furthermore, we observed that several functional hallmarkers exhibited significant correlation with the four potential diagnostic biomarkers (Fig. 8B). TNF-α signaling via NF-κB, P53 pathway, TGF-β signaling, inflammatory response, KRAS signaling, IL-6-JAK-STAT3 signaling, hypoxia, IL-2-STAT5 signaling, and angiogenesis, etc. were significantly positively correlated with BCL2L11, whereas significantly negatively correlated with NAGS, HDHD3, and RMND1. Adipogenesis, bile acid metabolism, DNA repair, E2F targets, fatty acid metabolism, oxidative phosphorylation, and peroxisome were significantly positively correlated with NAGS, HDHD3, and RMND1, whereas significantly negatively correlated with BCL2L11.

### 3.7 MRGs-based diagnostic nomogram

**Fig. 9:**
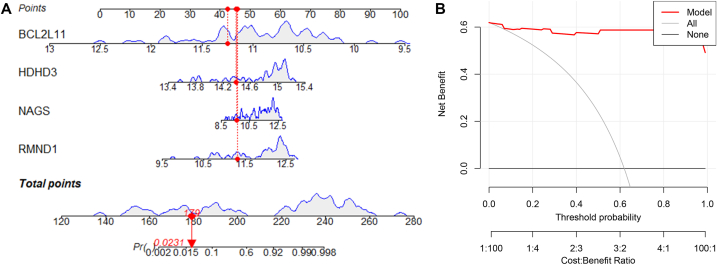
Construction of the diagnostic nomogram. **(A)** A nomogram is constructed based on the four MRGs. **(B)** Analysis of decision curves.

The four potential diagnostic biomarkers (BCL2L11, NAGS, HDHD3, and RMND1) were applied to construct the diagnostic nomogram (Fig. 9A). BCL2L11 was determined to be a protective predictor for NAFLD, while NAGS, HDHD3, and RMND1 were determined as risky factors for NAFLD. The DCA revealed that the diagnostic nomogram has obvious clinical benefit at certain threshold probability, indicating the actual advantage of the diagnostic model in clinical benefit (Fig. 9B).

### 3.8 Expression of MRGs in NAFLD

Compared to healthy participants, BCL2L11 mRNA expression was significantly downregulated, while the mRNA levels of NAGS, HDHD3, and RMND1 were significantly altered in peripheral blood of NAFLD patients (*P* < 0.01) (Supplementary Fig. 1A). Furthermore, we established a NAFLD model by HFD, which resulted in a significant increase in the body weight and liver weight of the mice in comparison to the control group. (Supplementary Figs. 1B–C). Pathological morphological changes in liver tissue were observed using HE and ORO staining. NAFLD mice showed higher NAS scores and ORO staining area (Supplementary Figs. 1D–E). The levels of serum TC, TG, AST, and ALT in NAFLD mice were also significantly increased (Supplementary Figs. 1F–G). Mitochondria play a major role in many cellular events, including the β-oxidation-induced breakdown of fatty acids, the generation of reactive oxygen species (ROS), and the production of metabolites in the tricarboxylic acid cycle [12]. We found that the MDA content in NAFLD mice was higher, and the activity of SOD and GSH-Px were significantly lower in comparison with the control group(*P* < 0.01) (Supplementary Fig. 1H).

**Fig. 10:**
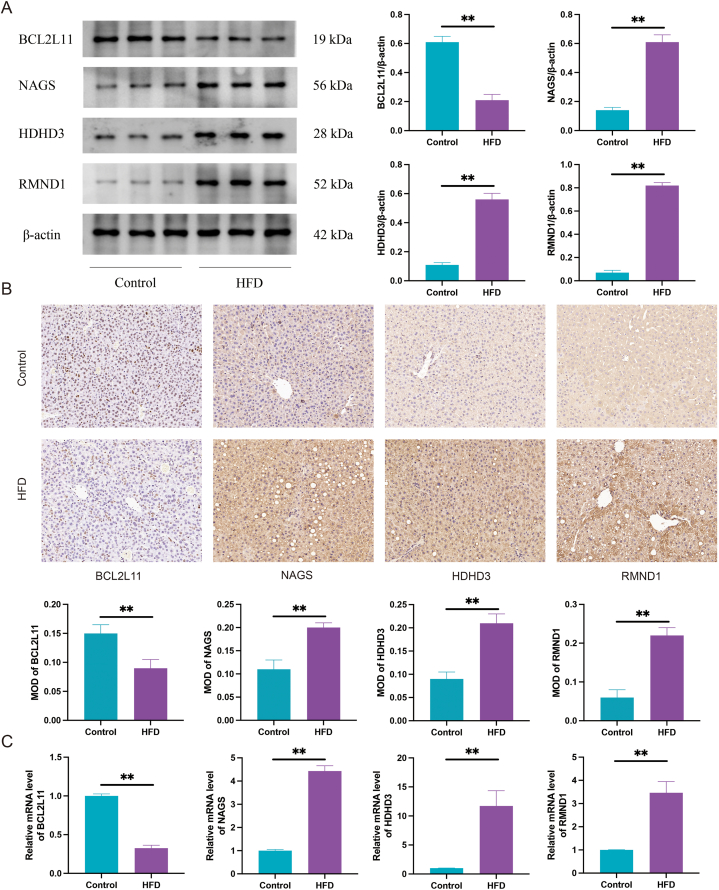
Expression of four potential diagnostic biomarkers in NAFLD mice. **(A)** Using Western blotting, the expression levels of four potential diagnostic biomarkers were tested in mouse liver tissue. **(B)** Immunohistochemistry is used to detect the expression levels of four potential diagnostic biomarkers in mouse liver tissue. **(C)** Using qRT-PCR, the levels of the four potential diagnostic biomarkers were determined in mouse liver tissue.

In addition, the expression of BCL2L11, NAGS, HDHD3 and RMND1 was validated in the liver tissue of NAFLD mice. The Western blot and IHC analysis results revealed that the expression of BCL2L11 in the liver tissue of HFD induced NAFLD mice was significantly downregulated, while the expression of NAGS, HDHD3, and RMND1 proteins was significantly upregulated (*P* < 0.01) (Fig. 10A and B). In addition, the results of qRT-PCR showed the same trend with Western blot and immunohistochemistry (Fig. 10C).

## 4 Discussion

NAFLD is predicted to become the most common indication for liver transplantation by 2030 and is the leading cause of liver-related morbidity and mortality in both adults and children [13,14]. Due to the lack of effective diagnostic tools, thousands of patients remain undiagnosed and untreated [15]. The dysfunction of the mitochondria plays an essential role in the pathogenesis of NAFLD [16]. Mitochondria are dynamically active organelles that serve not only as the center of energy metabolism, but also as the major source of ROS production in the cell [17]. Hepatocytes, which are rich in mitochondria, oxidize dietary glucose and fatty acids through the β-oxidation and tricarboxylic acid (TCA) cycles. Impaired β-oxidation inhibits peroxisomal and cytochrome oxidation of FFA, resulting in significant increase in ROS and harmful by-products [18]. In healthy cells, redox molecules within the matrix of the mitochondria will neutralize most of the ROS by converting them into water. However, chronic activation of mitochondrial function in the presence of lipid overload can cause excessive electron leakage from complex I and III of the electron transport chain, leading to substantial oxidative stress in the liver [19,20]. In addition, elevated levels of ROS lead to prolonged opening of the mitochondrial permeability transition pore (mPTP), a pore through the mitochondrial membrane, which leads to mitochondrial depolarization, failure of oxidative phosphorylation, ATP depletion, and release of pro-apoptotic factors [21]. Furthermore, increased mPTP opening also leads to an osmotic imbalance that induces swelling of the mitochondrial matrix and leads to rupture of the outer mitochondrial membrane, processes that will lead to the destruction of the mitochondria and even the cell itself and will drive the progression of NAFLD [22]. However, few previous studies have indicated the potential diagnostic value of mitochondrial regulators in NAFLD. Therefore, our primary aim was to investigate the feasibility of using mitochondrial regulators for diagnostic prediction in NAFLD.

In the present study, 34 NAFLD samples were divided into two clusters by consensus clustering analysis based on key mitochondrial regulators, with 25 NAFLD samples included in cluster 1 and 14 NAFLD samples included in cluster 2. The two subtypes did not show significant differences in clinical characteristics due to limited clinical information on NAFLD samples. Therefore, NAFLD may be a disease with two subtypes, and further clinical studies are needed to confirm whether the two molecular subtypes have different clinical features or prognoses. Nevertheless, we identified the differences between these NAFLD subtypes by immune cell infiltration analysis, such as the high mast cell subtype and high γδ T cell subtype. In addition, most NAFLD patients belong to the high γδ T cell subtype, and therapeutic targeting of γδ T lymphocytes may become an essential approach in NAFLD, as confirmed by recent studies [[23], [24], [25]].

Ultrasound has been suggested as a first-line screening tool to define steatosis in specific populations, whereas the diagnosis of NAFLD requires the exclusion of other causes of chronic liver disease or other causes of steatosis. Liver biopsy remains the current gold standard for diagnosis, despite limitations in terms of sampling variability, invasiveness and high cost [26]Subsequently, we obtained four potential diagnostic biomarkers for NAFLD by overlapping the results of SVM-RFE, LASSO, and RF machine learning models. Compared to the overfitting of RF and the complex parameter tuning process of SVM-RFE and LASSO, the use of a combination of these three machine learning methods allows modeling and feature selection of the data from different perspectives and improves the robustness of the model. Although a number of machine learning studies on diagnostic markers for NAFLD already exist, however our study is the first to focus on mitochondrial regulatory factors due to the importance of mitochondrial disorders in NAFLD pathology [[27], [28], [29], [30], [31], [32], [33]]. No previous study has indicated the diagnostic value of BCL2L11, NAGS, HDHD3 and RMND1 in NAFLD, so we reported for the first time that the four MRGs may serve as promising diagnostic biomarkers for NAFLD, whether alone or in combination. This is an easy method which can be tested by peripheral blood.

BCL2 like 11 (BCL2L11), also known as BIM, locates in the outer membrane of mitochondria and functions as a facilitator of mitochondrial apoptosis [34,35]. Mota et al. [36] summarized that several signaling pathways including BCL2L11-mediated mitochondrial apoptosis were enhanced by lipotoxicity and glucotoxicity to accelerate hepatocellular apoptosis in NAFLD. N-acetylglutamate synthase (NAGS) is a mitochondrial enzyme that catalyses the formation of N-acetylglutamate from glutamate and acetyl-coenzyme A and is involved in the regulation of the urea cycle in hepatocytes [37,38]. Haloacid dehalogenase like hydrolase domain containing 3 (HDHD3) is a hydrolase in mitochondria, which has been identified as a mitochondrial protein involved in maintaining cellular energy [39]. HDHD3 was found to be activated by protein kinase AMP γ 1 (PRKAG1), PRKAG2, and ATP production are involved. A previous study suggested that the morphology of mitochondria in mice hepatocytes can be preserved via upregulation of HDHD3, which was relevant to energetic homeostasis [40].Which more, HDHD3 has also been demonstrated to be significantly up-regulated in renal tubules of streptozotocin induced alpha-2u globulin nephropathy [41]. Required for meiotic nuclear division 1 homolog (RMND1) is reported to be a pivotal player in mitochondrial transcription and its mutation may contribute to multiple diseases like Perrault syndrome, leukoencephalopathy, and severe renal hypoplasia [[42], [43], [44]].

Furthermore, we noticed that the present study significantly activated three functional hallmarks (peroxisome, fatty acid metabolism, and bile acid biosynthesis) in NAFLD. The three functional hallmarkss were all significantly positively correlated with NAGS, HDHD3, and RMND1, while significantly negatively correlated with BCL2L11. The essence of NAFLD is chronic metabolic dysregulation in hepatocytes, including bile acid and fatty acid metabolism. This aetiological thinking also provides a therapeutic perspective for NAFLD [45]. The peroxisome proliferator-activated receptor (PPAR) is a type of nuclear receptor essential for regulating fatty acid metabolism and bile acid biosynthesis in hepatocytes [46]. Application of PPAR agonists, one of the anti-hyperglycaemic drug classes, has been proven to be effective in ameliorating NAFLD, as well as hyperlipidemia [47]. Thus, the three potential diagnostic biomarkers, NAGS, HDHD3 and RMND1, may primarily contribute to NAFLD via involvement in aberrant fatty acid/bile acid metabolism. More experimental evidence is expected to entitle them to be applicable therapeutic targets for NAFLD. In addition, we also observed that the IL-6-JAK-STAT3 signaling pathway was significantly downregulated in NAFLD and significantly positively correlated with BCL2L11. Cai et al. [48] reported that enhancement of IL-6-induced STAT3 phosphorylation can significantly alleviate high-fat diet-related NAFLD, and insulin resistance in mice. Thus, the functional characterization of BCL2L11 may be highly relevant to the IL-6-JAK-STAT3 pathway in the pathogenesis of NAFLD, which requires further experimental verification.

In the present study, we internally validated the expression levels of BCL2L11, NAGS, HDHD3 and RMND1 in NAFLD of the GEO dataset. Meanwhile, we selected 30 NAFLD patients for validation, and the results of the study followed the same trend as the dataset, BCL2L11 expression was significantly downregulated, while NAGS, HDHD3 and RMND1 expression levels were significantly upregulated in NAFLD patients. Subsequently, we established a NAFLD mouse model induced by a high-fat diet, which is currently recognized as a model of NAFLD with the same pathological findings as humans, and is able to better mimic the metabolic profile of NAFLD, with symptoms such as obesity, visceral adiposity, hyperglycemia, hyperinsulinemia, hyperleptinemia, glucose intolerance, oxidative stress, and insulin resistance; however, the degree of liver injury was milder in the high-fat diet model alone compared with that of MCD [49]. However, compared to the MCD diet model, the high-fat diet alone model showed less liver injury and a greater strain dependence on the extent of lesions. The results of histopathology, serum enzymology and lipid levels showed significant NAFLD features and high levels of oxidative stress (increase in MDA content, decrease in GSH-Px content and decrease in SOD activity). Our results in the liver also confirmed that BCL2L11 expression was significantly downregulated, while NAGS, HDHD3 and RMND1 expression levels were significantly upregulated in NAFLD.

This study also has some limitations. First, the present study only relies on transcriptomic data, which provide only a limited snapshot of mitochondrial dysfunction. This may need to be combined with multi-omics data and thus analyzed in a comprehensive manner. Second, static gene expression markers may not fully capture the dynamic mitochondrial changes underlying NAFLD progression. Also, prognostic data linking biomarkers to outcomes such as fibrosis and cirrhosis are lacking. Finally, sample size for the validation cohort (30 NAFLD patients) is relatively small and that larger, independent cohorts are needed to confirm the diagnostic value of the four MRGs.

## 5 Conclusion

The present study established a molecular model for the diagnosis of NAFLD based on mitochondrial regulators, providing new insights into the diagnosis and potential treatment of NAFLD. Their role in the mechanisms of NAFLD, particularly immune dysregulation, may be the most important area for future research.

## Data availability statement

Data will be made available on request, including R and other custom scripts for analyzing.

## Ethics statement

The research protocols were performed with the approval of Clinical Ethics Committee of Heilongjiang Academy of Traditional Chinese Medicine (No.2022-003-01). All procedures were performed strictly in compliance with the Declaration of Helsinki 1964 or equivalent ethical principles. All patients involved in this study had given their informed consent before study.

All of the experimental procedures involving animals were conducted in accordance with the Animal Care Guidelines of Heilongjiang Academy of Traditional Chinese Medicine, China and approved by the Administration Committee of Experimental Animals of Heilongjiang Academy of Traditional Chinese Medicine (No. SY3R-2022035).

## Funding

This research was funded by Excellent Youth Project of 10.13039/501100005046Heilongjiang Natural Science Foundation (YQ2022H015) and Projects of Heilongjiang Administration of Traditional Chinese Medicine (ZHY2020-041).

## CRediT authorship contribution statement

**Bingyu Wang:** Conceptualization. **Hongyang Yu:** Writing – review & editing, Writing – original draft. **Jiawei Gao:** Formal analysis, Data curation. **Liuxin Yang:** Methodology, Investigation. **Yali Zhang:** Visualization, Validation, Software. **Xingxing Yuan:** Writing – review & editing, Writing – original draft, Resources, Funding acquisition. **Yang Zhang:** Conceptualization.

## Declaration of competing interest

The authors declare that they have no known competing financial interests or personal relationships that could have appeared to influence the work reported in this paper.

## Figures and Tables

**figs1:**
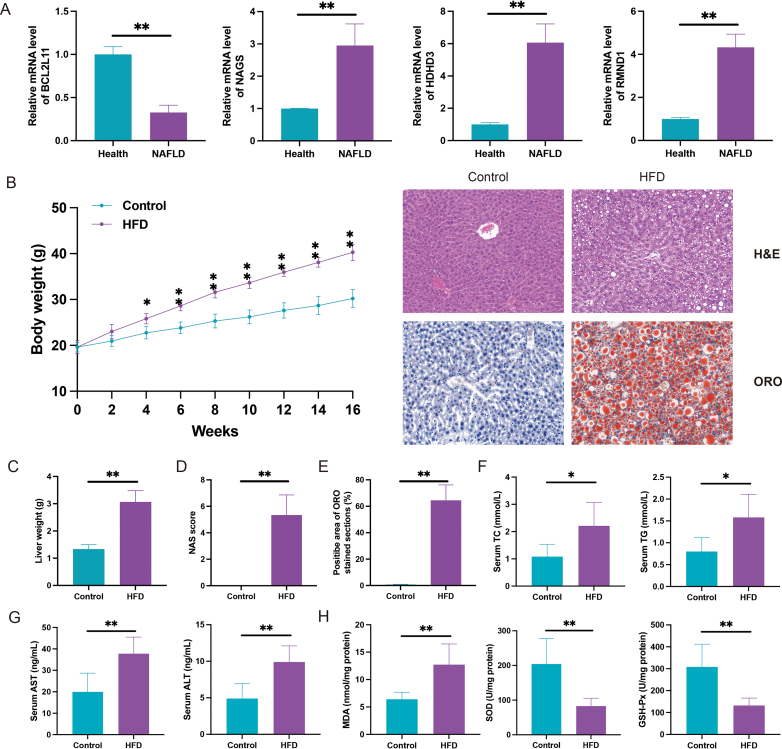


**figs2:**
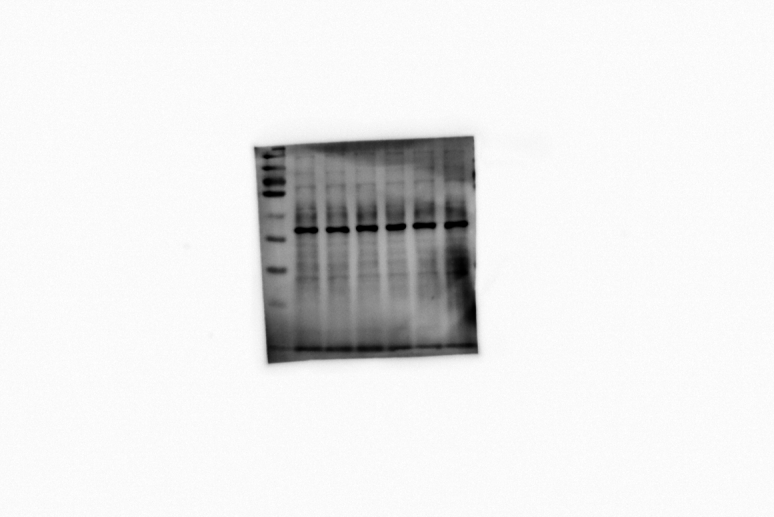


**figs3:**
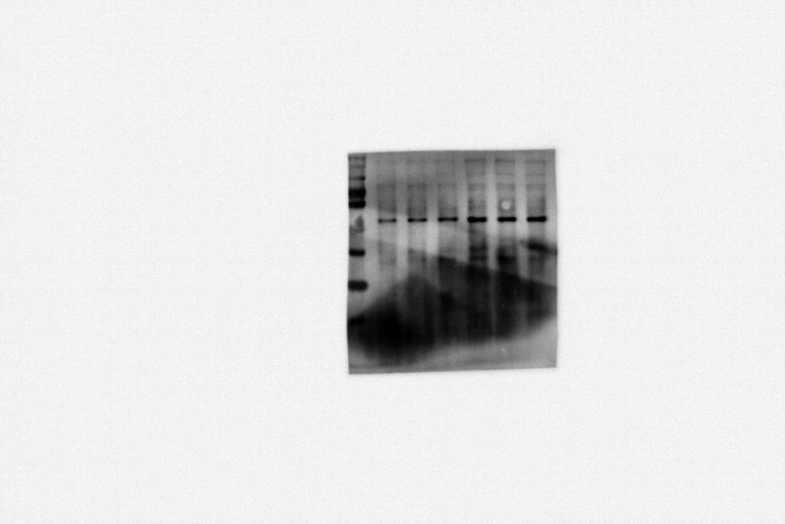


**figs4:**
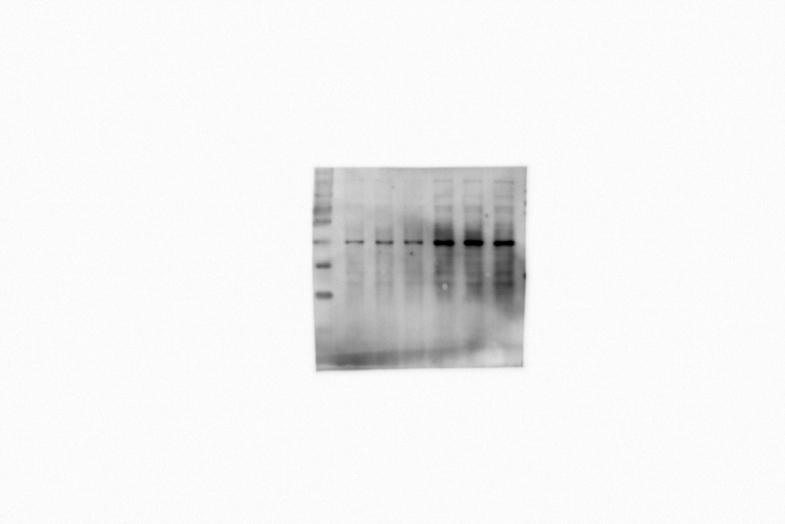


**figs5:**
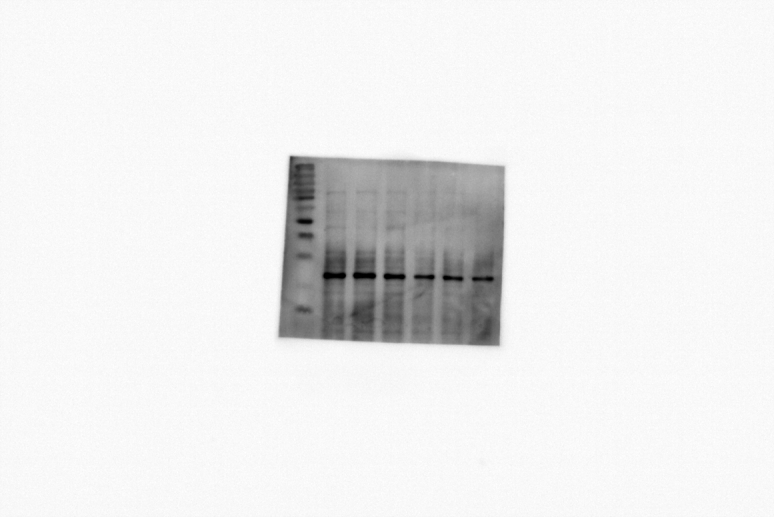


**figs6:**