# Supplementary material for: Machine learning deciphers the significance of mitochondrial regulators on the diagnosis and subtype classification in non-alcoholic fatty liver disease
Source: Heliyon. 2024 Apr 23;10(9):e29860. doi: 10.1016/j.heliyon.2024.e29860 (PMC11066337; doi:10.1016/j.heliyon.2024.e29860)
Supplement: Multimedia component 1 [file mmc1.docx]

**
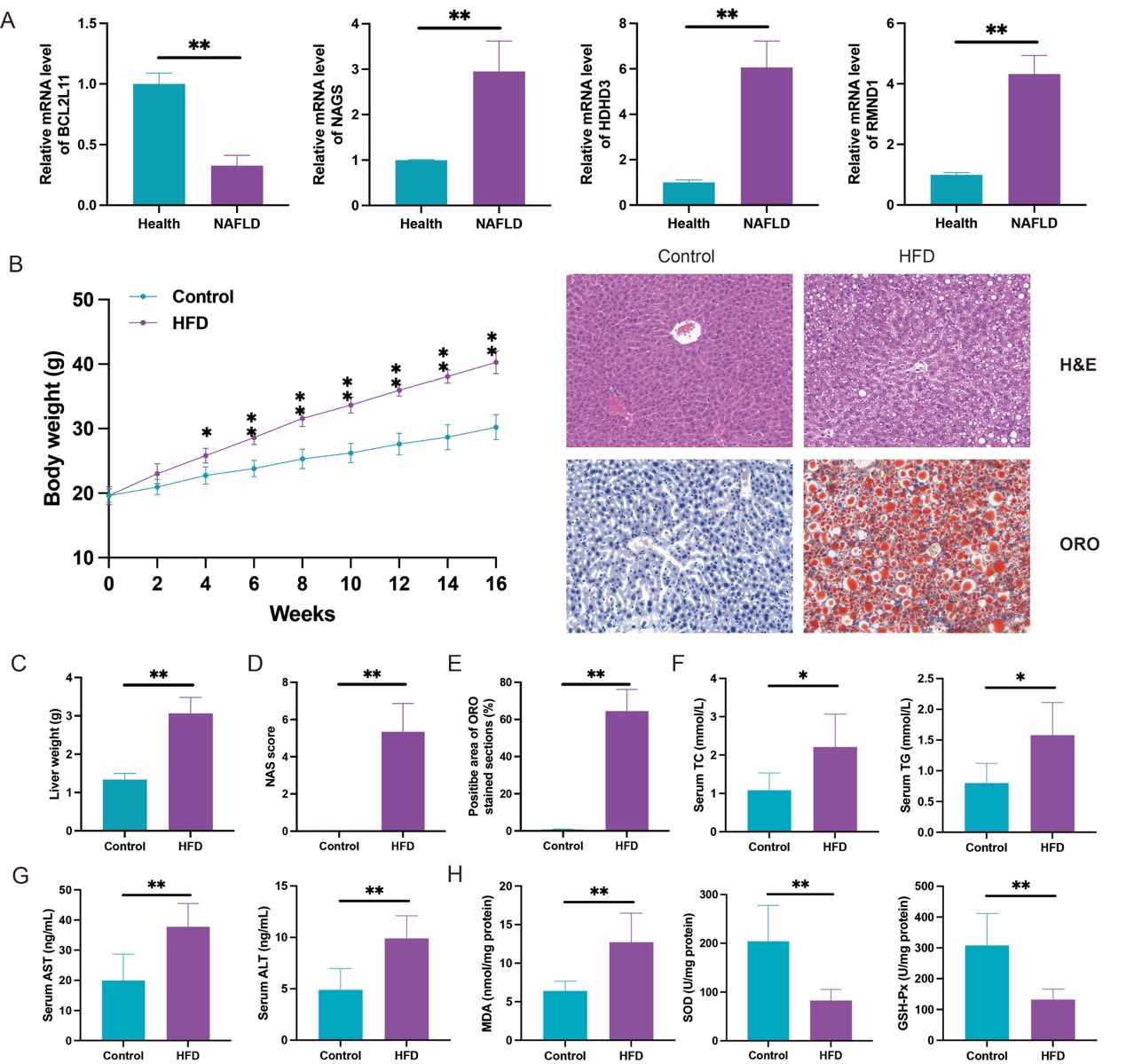
**

**Supplementary Figure 1**. Expression of four potential diagnostic biomarkers in NAFLD patients and construction of a NAFLD mouse model. **(A)** Expression levels of four potential diagnostic biomarkers in NAFLD patients were detected by qRT-PCR. **(B)** Monitor the body weight for 16 weeks. **(C)** Comparison of liver weight between control group and HFD group mice. **(D-E)** Comparison of NAFLD activity score and ORO positive staining area in mouse liver tissue. **(F)** Comparison of the lipid levels in serum. **(G)** Comparison of liver function levels in mouse serum. **(H)** Comparison of oxidative stress indicators in mouse liver tissue.

**1. Diagnostic criteria**

According to the diagnostic criteria of the 2018 updated "Guidelines for the Diagnosis and Treatment of Nonalcoholic Fatty Liver Disease" by the Chinese Medical Association:

1. No history of alcohol use or alcohol intake of less than 70 g/week for men and less than 140 g/week for women.
2. With the exception of certain diseases that can lead to NAFLD, such as hepatitis B and C virus, autoimmune liver disease, drug-induced liver disease, genetic metabolic disease, etc.
3. Symptoms and signs such as fatigue, indigestion, guarding pain around the liver, and hepatosplenomegaly are present in addition to the primary disease.
4. Diagnostic criteria for diffuse fatty liver disease are consistent with imaging features of the liver.

**2. Inclusion criteria**

1. Meets diagnostic criteria for NAFLD.
2. Age between 18 and 65 years.
3. Consent to participate in the study.

**3. Exclusion criteria**

1. Patients with concomitant alcoholic liver disease, viral liver disease, autoimmune liver disease, or hereditary liver disease.
2. Organ failure.
3. Other serious diseases, such as the heart, liver, kidneys, hematopoietic system, mental illness, and cancer.
4. Pregnant and lactating women.
5. Recent trauma, surgery, or other situations that have led to increased stress response in the past three months.
6. Incomplete information.

**Supplementary Table 1. Characteristics of the participants**

| Characteristics | NAFLD | Healthy volunteer | *P* value |
| --- | --- | --- | --- |
| No. of participants | 30 | 30 |  |
| Males (n) | 13 | 15 | 0.605 |
| Females (n) | 17 | 15 |  |
| Age (years) | 35.60±4.85 | 34.33±6.59 | 0.400 |
| BMI (Kg/m^2^) | 23.66±1.67 | 22.16±1.44 | ＜0.001 |
| WC (cm) | 82.45±3.71 | 76.15±3.83 | ＜0.001 |
| TC (mmol/L) | 5.69±0.68 | 4.60±0.56 | ＜0.001 |
| TG (mmol/L) | 2.14±0.37 | 1.41±0.18 | ＜0.001 |
| LDL-C (mmol/L) | 2.83±0.27 | 2.59±0.20 | ＜0.001 |
| HDL-C (mmol/L) | 1.49±0.31 | 1.71±0.37 | 0.014 |
| FBG (mmol/L) | 5.23±0.50 | 5.06±0.39 | 0.145 |
| ALT (U/L) | 46.23±23.61 | 22.17±7.64 | ＜0.001 |
| SBP (mmHg) | 118.63±8.39 | 116.73±6.48 | 0.330 |
| DBP (mmHg) | 73.83±5.35 | 71.07±4.94 | 0.042 |
| Smoking status (%) |  |  |  |
| Current smoker | 2 | 4 | 0.6716 |
| Ex-smoker | 7 | 6 | 0.5082 |
| Non-smoker | 21 | 20 | 0.500 |
| Alcohol drinking status |  |  |  |
| Everyday | 1 | 0 | 1.000 |
| Sometime | 12 | 10 | 0.395 |
| Ex-drinker | 7 | 4 | 0.253 |
| Non-drinker | 10 | 10 | 1.000 |
| Education level (≥college, %) | 14(46.7%) | 18(60.0%) | 0.219 |
| Family history of disease (%) |  |  |  |
| Cardiovascular disease | 21 | 18 | 0.294 |
| Hypertension | 22 | 18 | 0.206 |
| Hyperlipidemia | 19 | 15 | 0.217 |
| Diabetes | 4 | 3 | 0.500 |

**Supplementary Table 2．The primer sequences were used in this study**

| **Species** | **Genes** | **Forward primer** | **Reverse primer** |
| --- | --- | --- | --- |
| Human | BCL2L11 | CAAGAGTTGCGGCGTATTGGAG | ACACCAGGCGGACAATGTAACG |
|  | NAGS | CAGTTCCAGACCTGCCATCACT | ATGTCCATGCGCTGCAAGAAGG |
|  | HDHD3 | TGTCCAGGATGCTCAGGCTGTA | AGATCACTGCCAGTCTCAGACC |
|  | RMND1 | GAGAAAACCTGGAAGGACTTTACG | AAGCGGAGTGCCCTCTTCTCAT |
|  | GAPDH | GTCTCCTCTGACTTCAACAGCG | ACCACCCTGTTGCTGTAGCCAA |
| Mouse | BCL2L11 | GGAGATACGGATTGCACAGGAG | CTCCATACCAGACGGAAGATAAAG |
|  | NAGS | GCCTGCGGAATAACAGTCAGAAG | TCCACGATGAGCCGAATCTGCT |
|  | HDHD3 | TGAAGCTGGCAGTGGTCTCCAA | AAATCCGTGGGTCGGGTTTAGG |
|  | RMND1 | GGACAGAGCAAACCTGGAAGAG | CGCTTCTCATTGAGGTGATTCCG |
|  | GAPDH | CATCACTGCCACCCAGAAGACTG | ATGCCAGTGAGCTTCCCGTTCAG |
