# Supplementary material for: Machine learning deciphers the significance of mitochondrial regulators on the diagnosis and subtype classification in non-alcoholic fatty liver disease
Source: Heliyon. 2024 Apr 23;10(9):e29860. doi: 10.1016/j.heliyon.2024.e29860 (PMC11066337; doi:10.1016/j.heliyon.2024.e29860)
Supplement: Multimedia component 2 [file mmc2.docx]

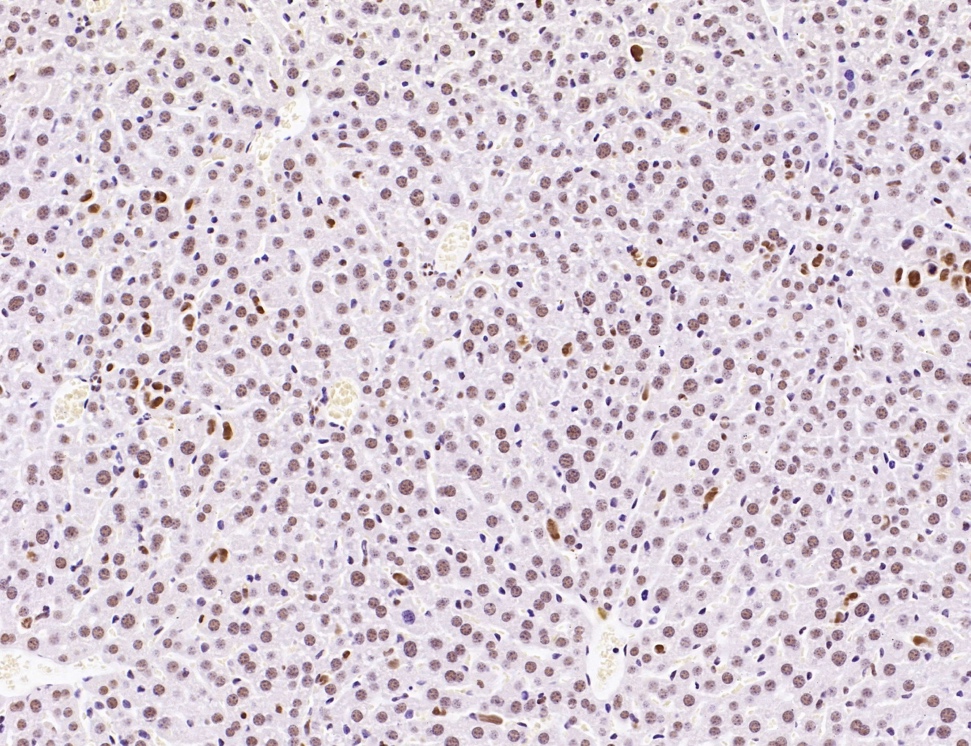


Control BCL2L1


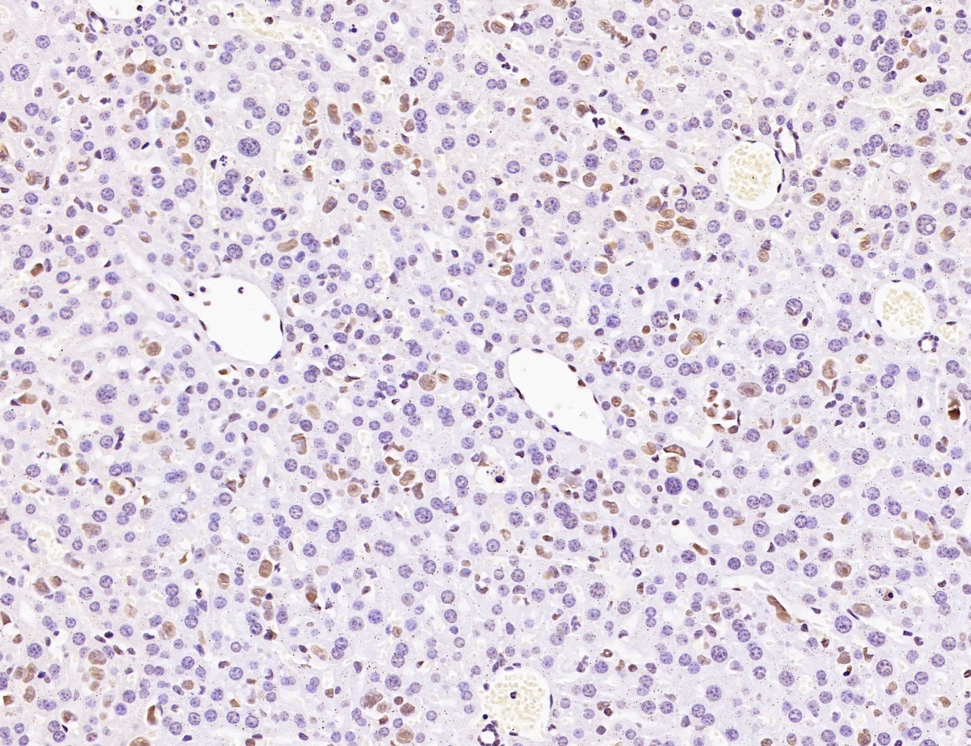


HFD BCL2L1


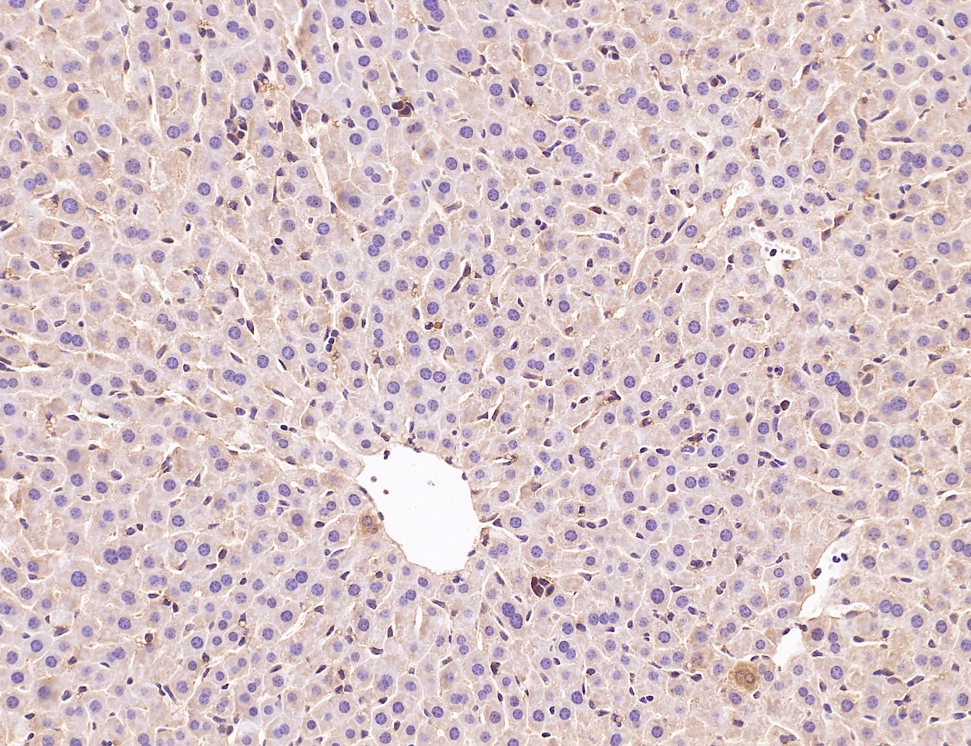


Control NAGS


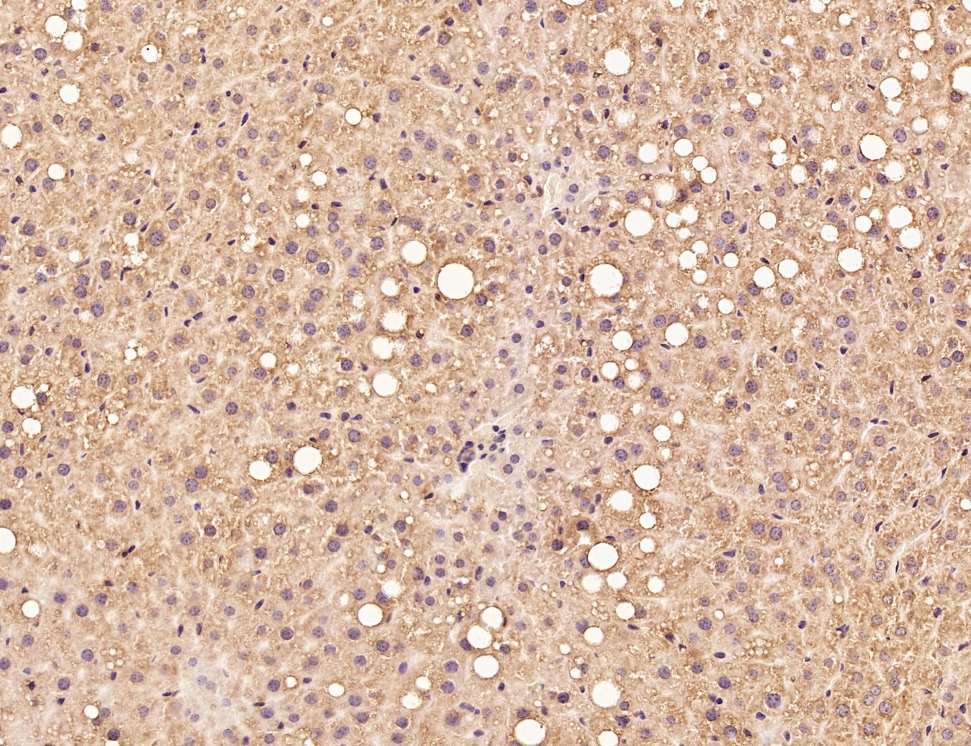


HFD NAGS


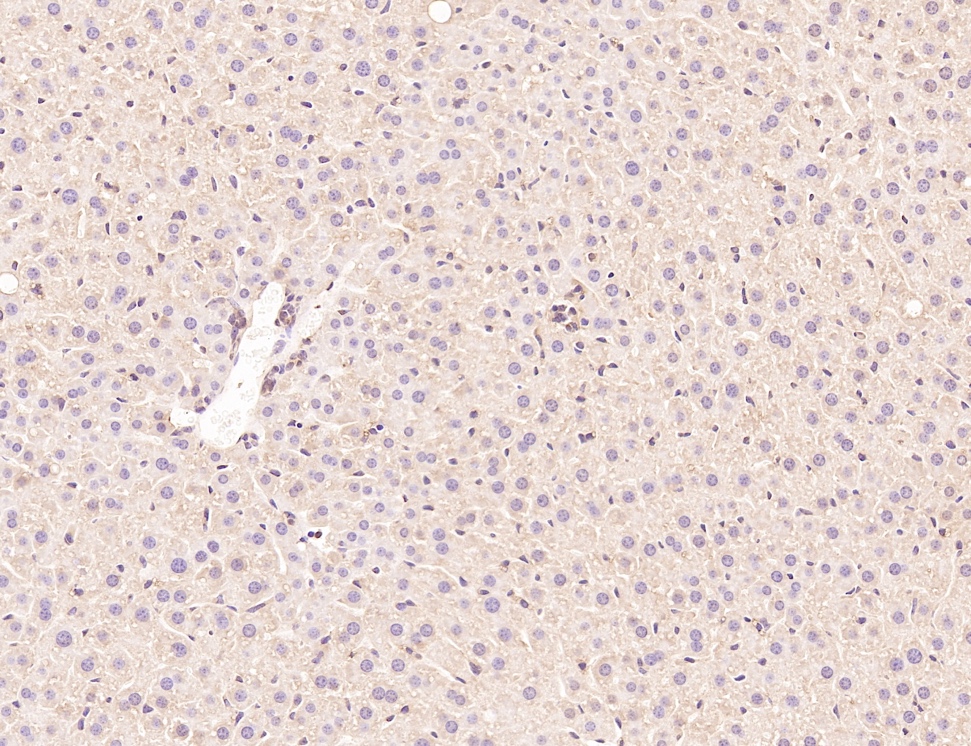


Control HDHD3


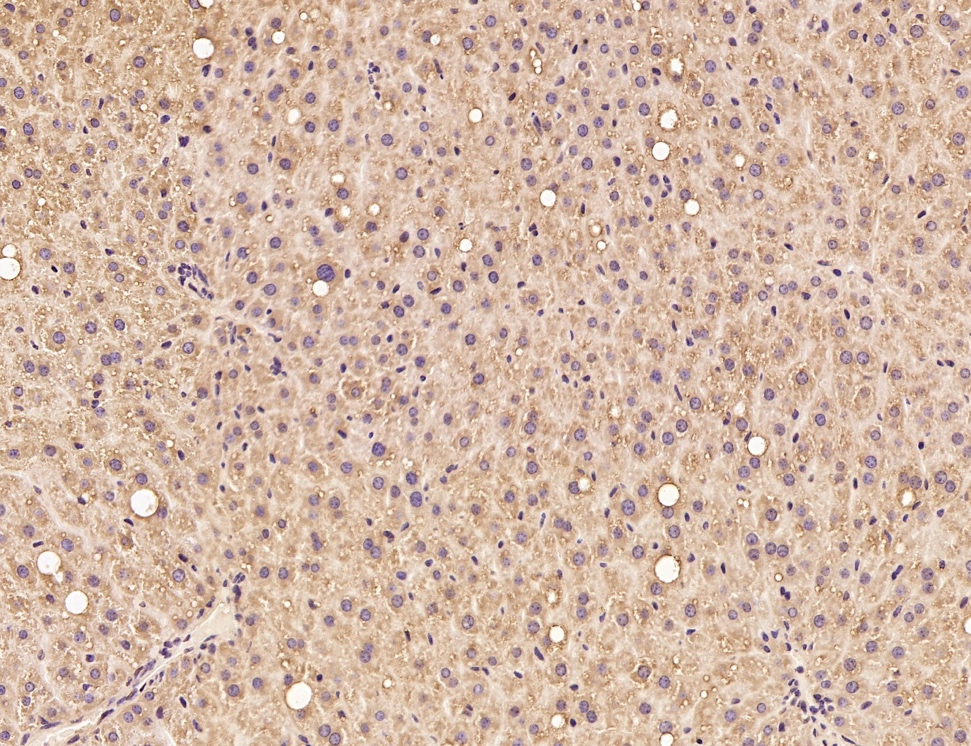


HFD HDHD3


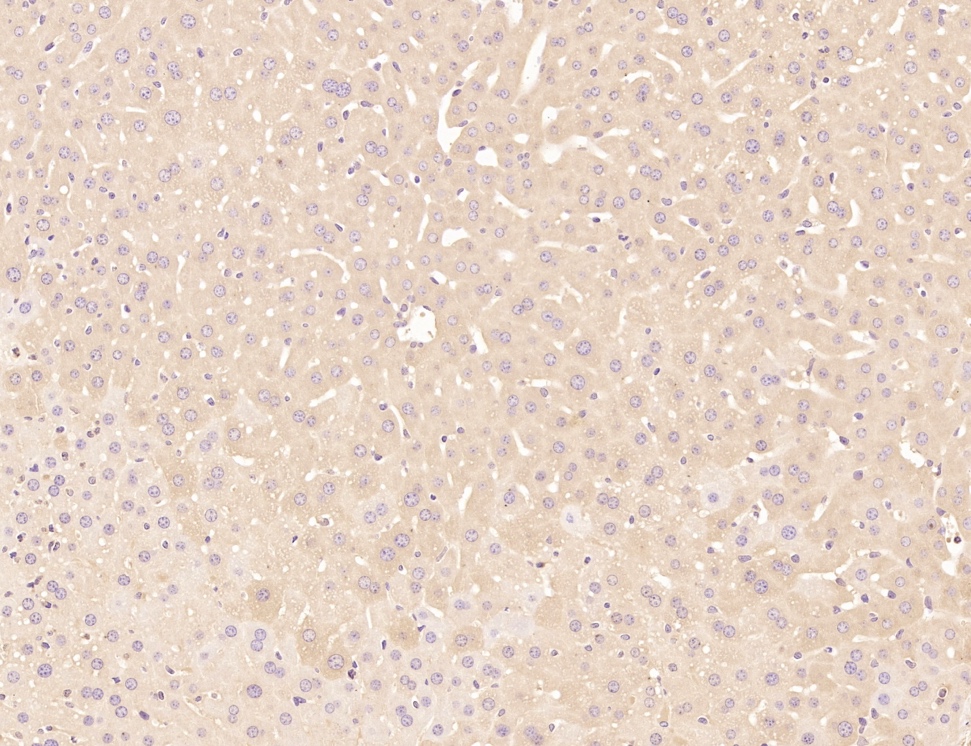


Control RMND1


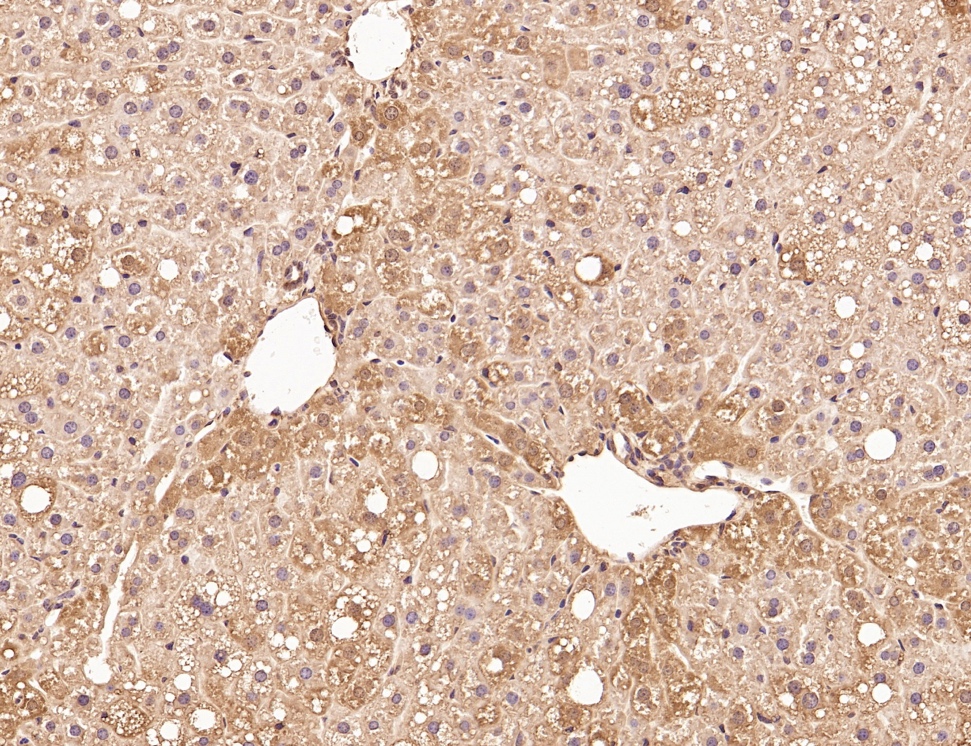


HFD RMND1


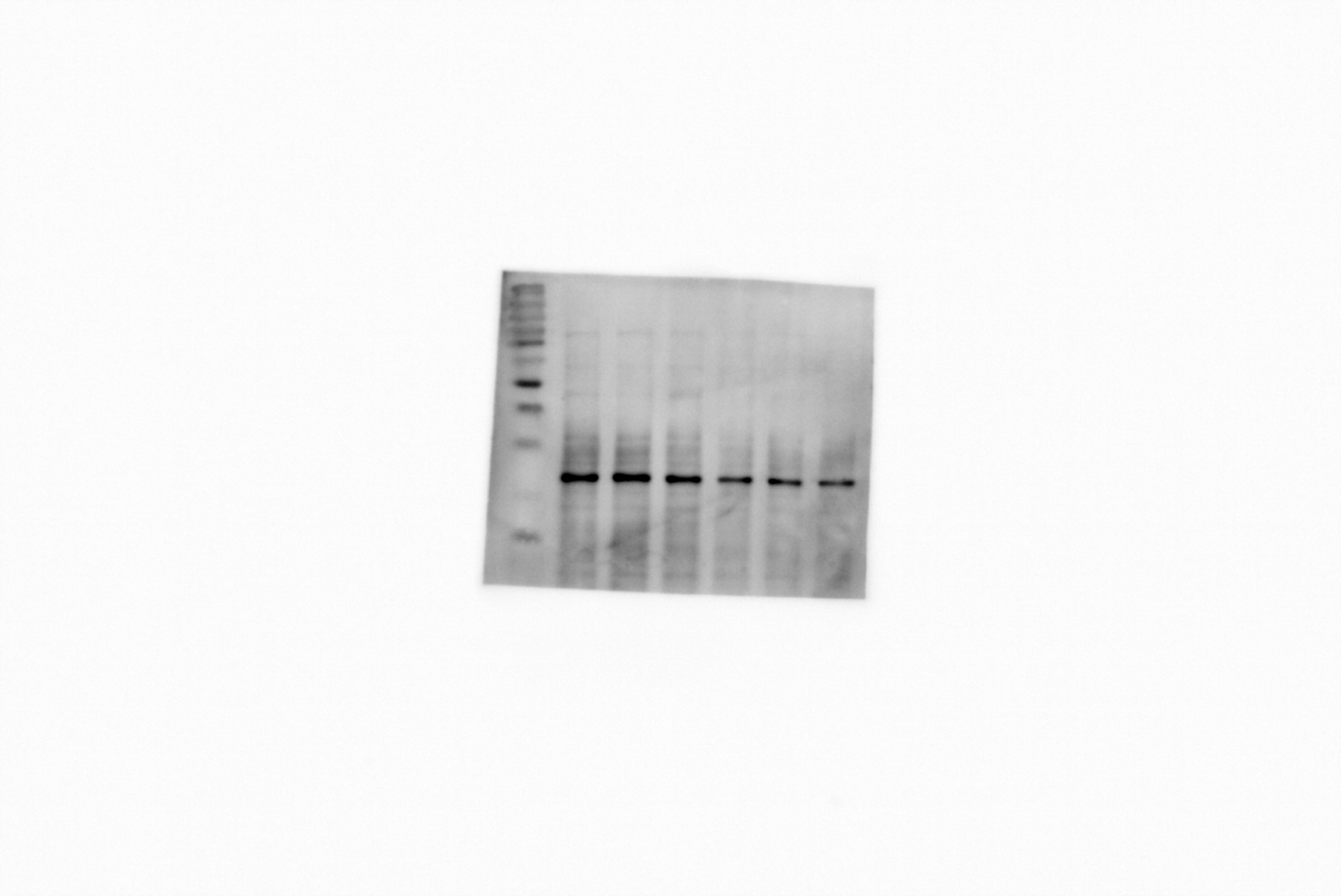


Control1

Control2

Control3

HDF1

HDF2

HDF3

13 kDa

BCL2L11 19 kDa


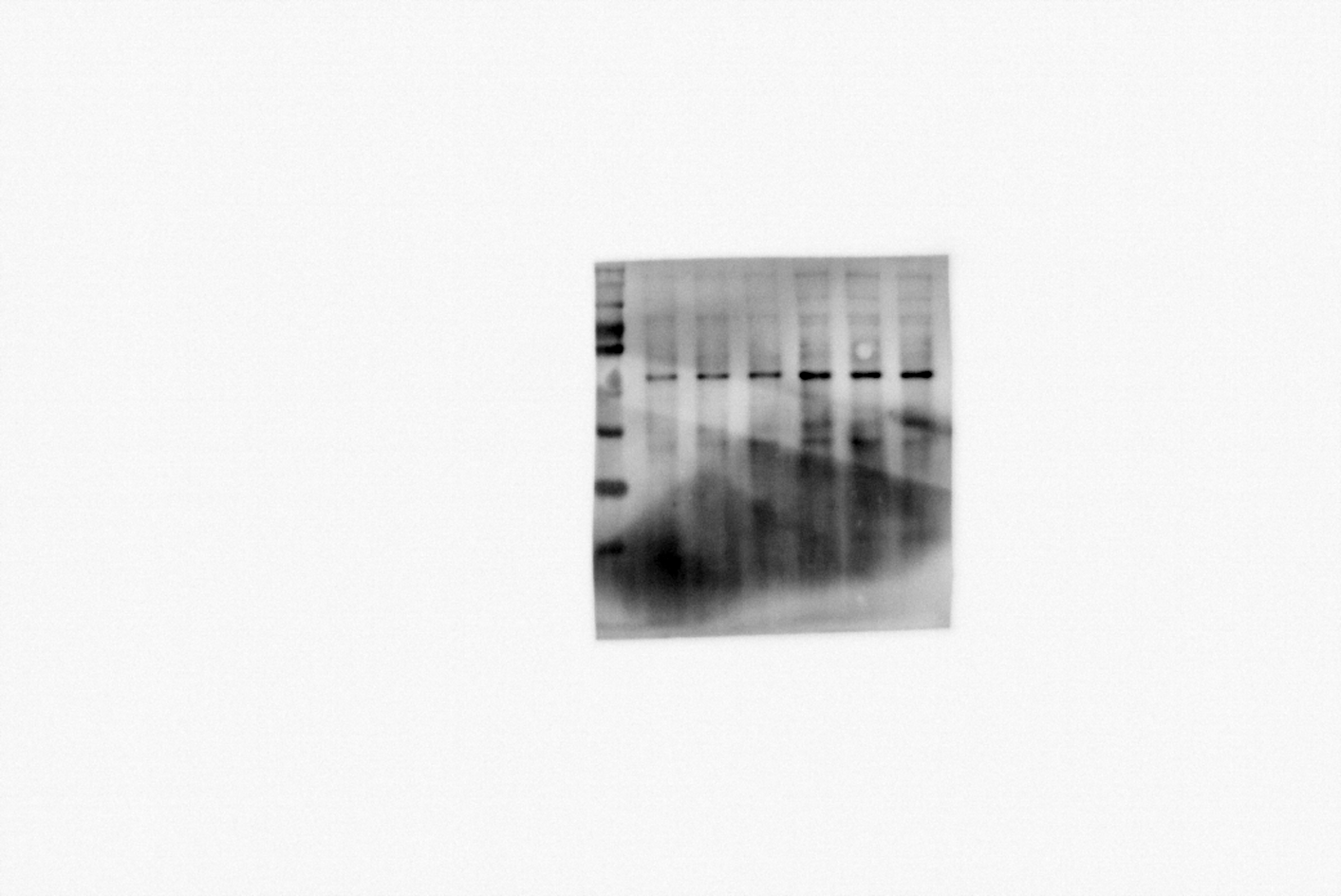


Control1

Control2

Control3

HDF1

HDF2

HDF3

50 kDa

NAGS 56 kDa


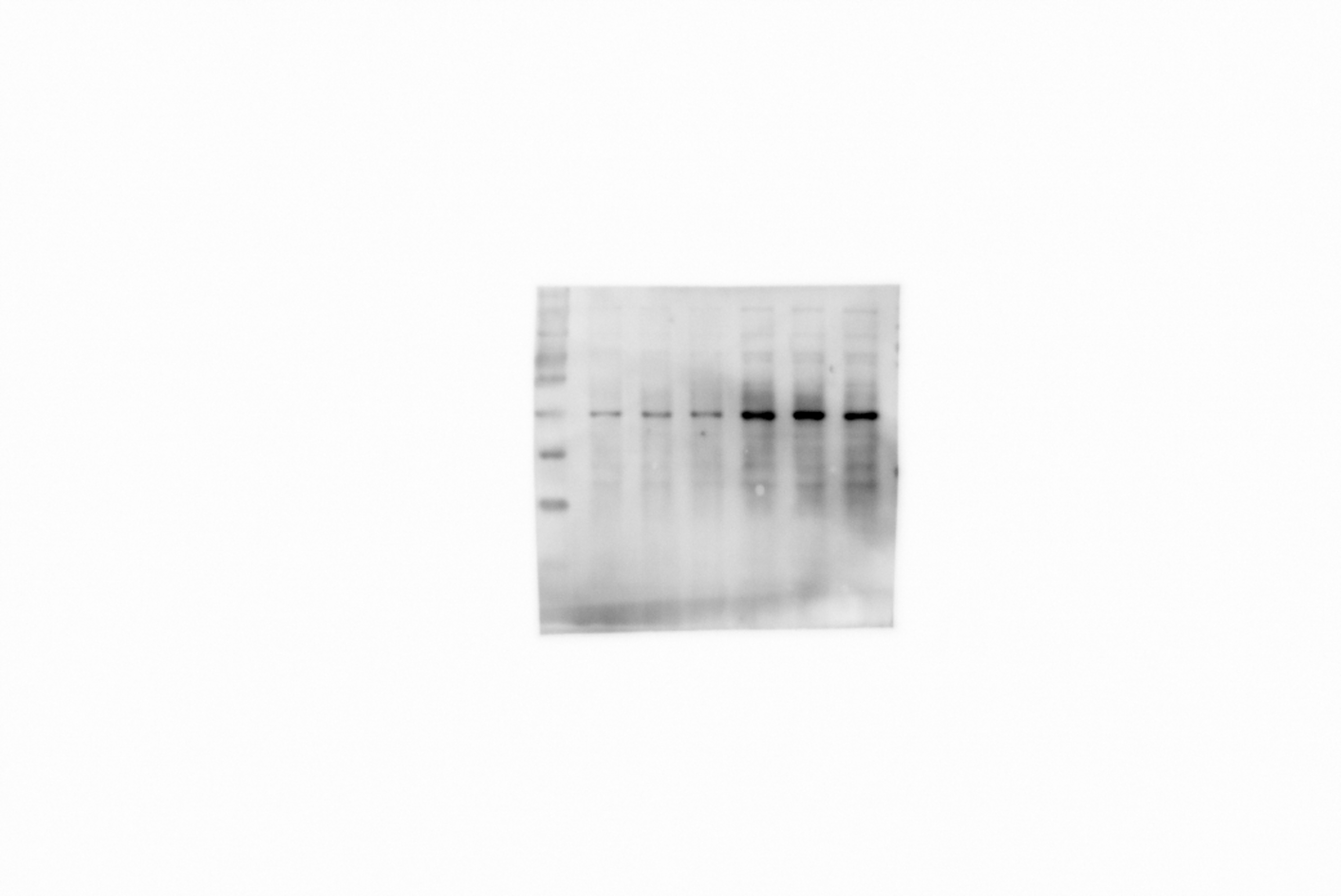


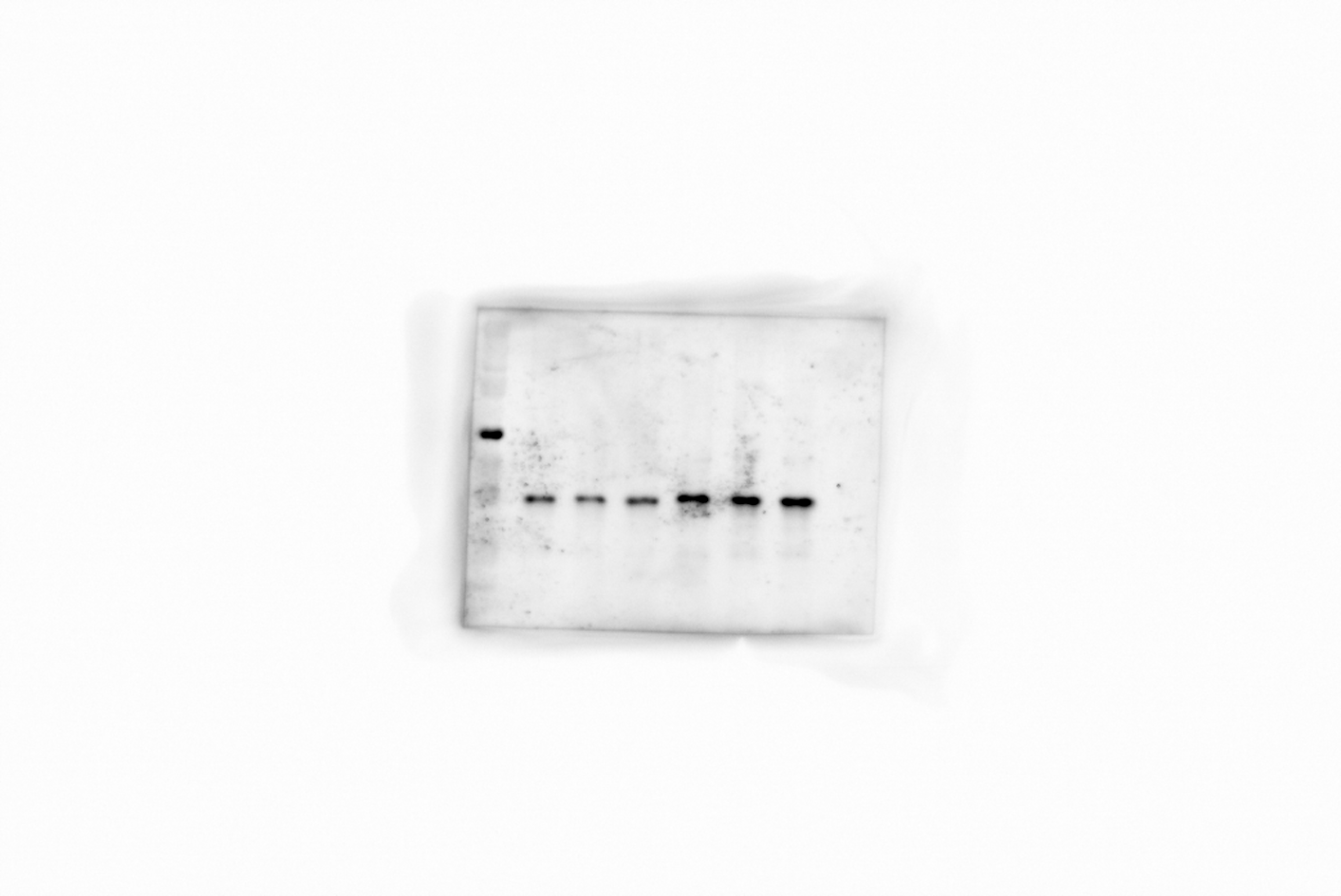


Control1

Control2

Control3

HDF1

HDF2

HDF3

30 kDa

HDHD3 28 kDa


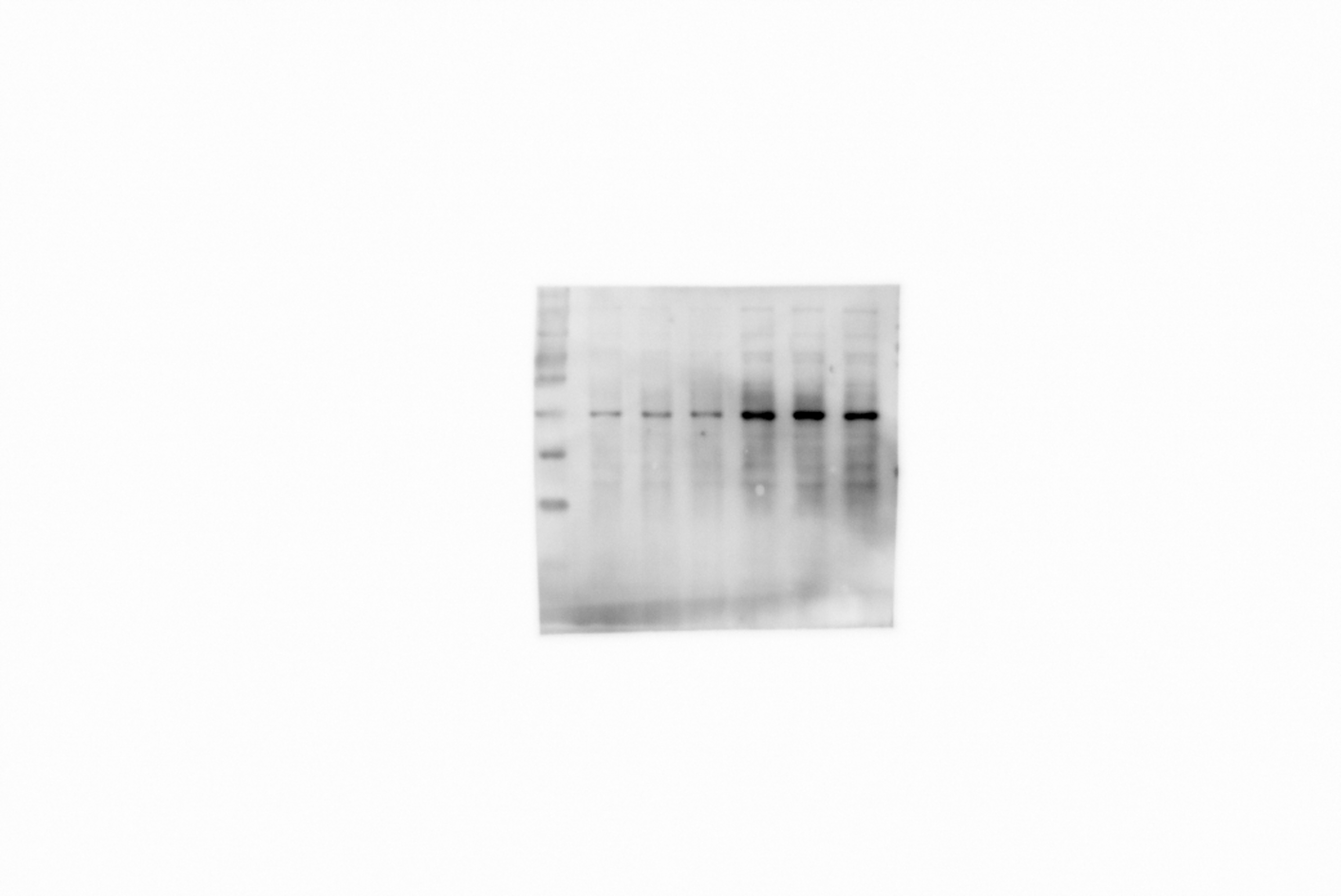


50 kDa

Control1

Control2

Control3

HDF1

HDF2

HDF3

RMND1 52 kDa


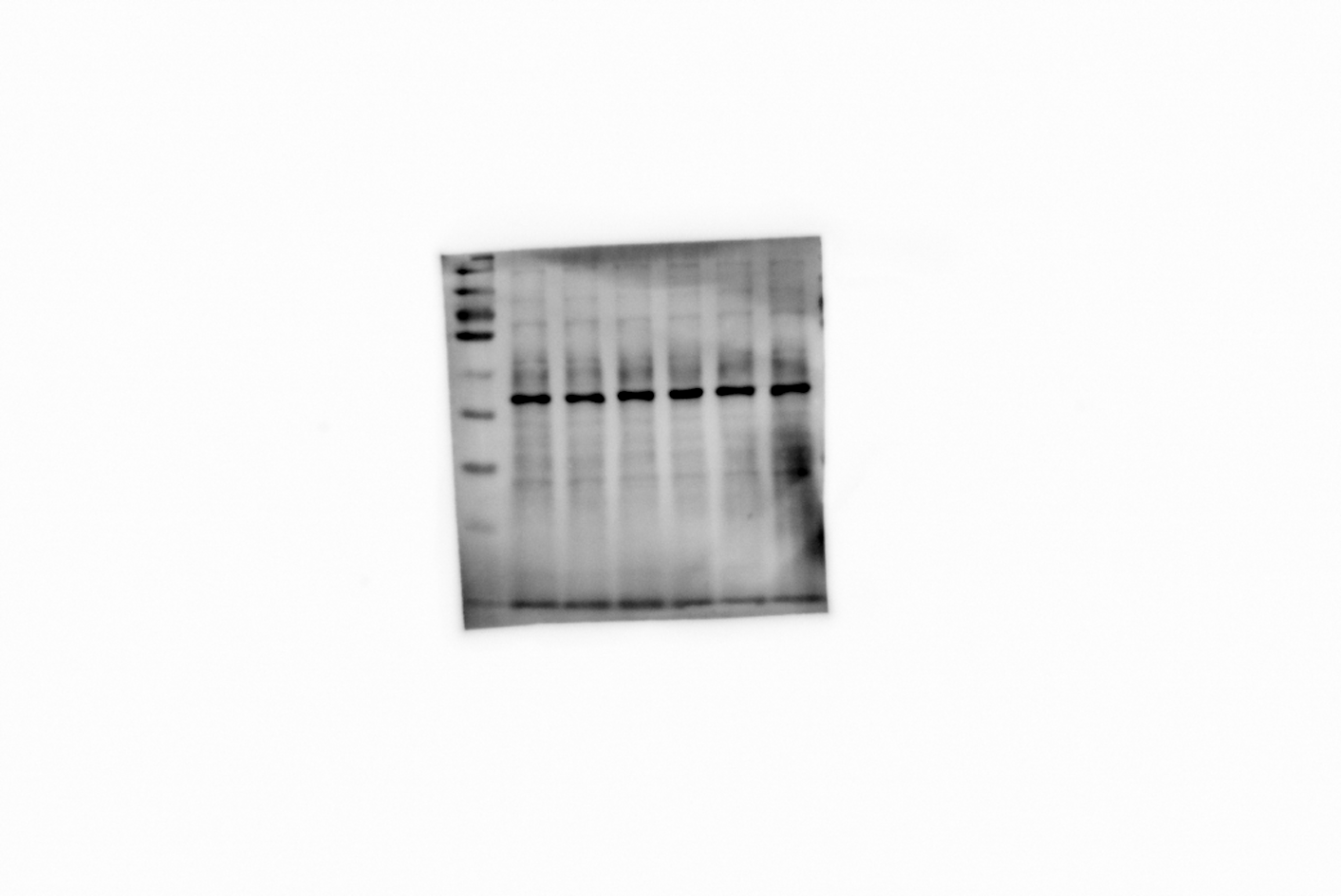


40 kDa

Control1

Control2

Control3

HDF1

HDF2

HDF3

β-actin 42 kDa
